# Direct and ancillary benefits of ecosystem‐based fisheries management in forage fish fisheries

**DOI:** 10.1002/eap.2421

**Published:** 2021-08-30

**Authors:** James N. Sanchirico, Timothy E. Essington

**Affiliations:** ^1^ Department of Environmental Science and Policy University of California, Davis Davis California 95616 USA; ^2^ University Fellow Resources For the Future Washington D.C. 20036 USA; ^3^ School of Fishery Science University of Washington Seattle Washington 01267 USA

**Keywords:** bioeconomics, natural resource management, optimal control, pseudo‐spectral numerical techniques

## Abstract

Natural resource management is evolving toward holistic, ecosystem‐based approaches to decision making. The ecosystem science underpinning these approaches needs to account for the complexity of multiple interacting components within and across coupled natural–human systems. In this research, we investigate the potential economic and ecological gains from adopting ecosystem‐based approaches for the sardine and anchovy fisheries off of the coast of California, USA. Research has shown that while predators in this system are likely substituting one forage species for another, the assemblage of sardine and anchovy can be a significant driver of predator populations. Currently, the harvest control rules for sardine and anchovy fisheries align more with traditional single species framework. We ask what are the economic and ecological gains when jointly determining the harvest control rules for both forage fish stocks and their predators relative to the status quo? What are the implications of synchronous and anti‐synchronous environmental recruitment variation between the anchovy and sardine stocks on optimal food‐web management? To investigate these questions, we develop an economic‐ecological model for sardine, anchovy, a harvested predator (halibut), and an endangered predator (Brown Pelican) that includes recruitment variability over time driven by changing environmental conditions. Utilizing large‐scale numerical optimal control methods, we investigate how the multiple variants of integrated management of sardine, anchovy, and halibut impact the overall economic condition of the fisheries and Brown Pelican populations over time. We find significant gains in moving to integrated catch control rules both in terms of the economic gains of the fished stocks, and in terms of the impacts on the Brown Pelican populations. We also compare the relative performance of current stylized catch control rules to optimal single species and optimal ecosystem‐based fisheries management (EBFM) across ecological and economic dimensions, where the former trade‐off considerable economic value for ecological goals. More generally, we demonstrate how EBFM approaches introduce and integrate additional management levers for policymakers to achieve non‐fishery objectives at lowest costs to the fishing sectors.

## Introduction

Natural resource management is evolving toward holistic, ecosystem‐based approaches to decision making to ensure the delivery of valued ecosystem goods and services (see, e.g., Brodziak and Link 2002, Pikitch et al. 2004). These approaches consider the multiple benefits that ecosystems provide, such as conservation of species, livelihoods, employment, cultural values, equity, and agency. They also seek to improve the scientific basis for decision making by highlighting trade‐offs among ecosystem services that emerge through alternative courses of management actions, with the goals of anticipating indirect consequences of actions on valued components of these systems and protecting components of systems that confer resilience. The ecosystem science underpinning these trade‐offs and indirect consequences, therefore, needs to account for the complexity of multiple interacting components within and across coupled natural–human systems (Marshall et al. 2018).

One primary area of research focus has been on the consequences of fishing in a food web context, either when fishing targets both predator and prey species (Larkin 1966, May et al. 1979), or when fishing targets species that otherwise play important ecological roles such as grazing coral reefs (Mumby et al. 2006) or are prey for species of conservation concern (Furness 2003). From an ecological standpoint, these considerations in management appear obvious: management that does not consider the linkages across food webs risks producing unintended consequences leading to coral reef degradation, lower ecosystem resilience, or increased extinction risk to protected species. Only recently have these considerations been posed in a more formal bioeconomic framework to ask when and where the economic *and* ecological gains from incorporating these considerations in decision making are greatest.

Two recent efforts shed light on this question. Kellner et al. (2011), for example, showed in a coral reef system that the incremental gains are greatest in cases when ecosystem approaches correct past fishery management failures and when multiple objectives (conservation and fishery management) are considered simultaneously. Asking a similar question, Essington et al. (2018) consider a series of alternative, nested economic‐ecological models that differ in terms of complexity of predator‐prey interactions of a common piscivore and prey fishery system, and showed that in many cases the cost of applying the wrong model (wrong in the sense that it is modeling incorrectly the predator–prey interaction) depends significantly on the state of the ecosystem. That is, if the predator is overfished while the prey is not and in the presence of strong depensation in the predator's population dynamics due to prey consuming their eggs, then the “wrong” model could lead to extinction of the predator population and significant reductions in the economic value from fishing both the prey and predator. On the other hand, if the populations of the predator and prey are relatively robust, then the gains from EBFM are modest, everything else being equal.

Both of the above efforts shed light on the question on when and where do economic gains accrue from EBFM, but did so via simple food webs and in did not consider whether these gains are upheld in more realistic environmental scenarios. The latter is particularly important, as the small pelagic fish that serve important roles both as fisheries targets and as critical prey for a number of marine predators (Pikitch et al. 2012) regularly undergo large oscillations in productivity (MacCall 2009).

In this research, we investigate the potential economic and ecological gains from adopting EBFM approaches for the sardine and anchovy fisheries off of the coast of California. Koehn et al. (2017) for example find that while predators in this system are likely substituting one forage species for another, the assemblage of sardine and anchovy can be a significant driver of predator populations (e.g., Brown Pelicans, salmon, and halibut). Currently, the harvest control rules for sardine and anchovy fisheries in the California current are determined in the more traditional single species management framework, i.e., the harvest rules for sardine do not consider the availability of anchovy as alternative prey, and vice versa. An open EBFM question is: what are the potential direct and indirect economic and ecosystem gains when jointly determining the harvest control rules for forage fish and their predators?

**Fig. 1:**
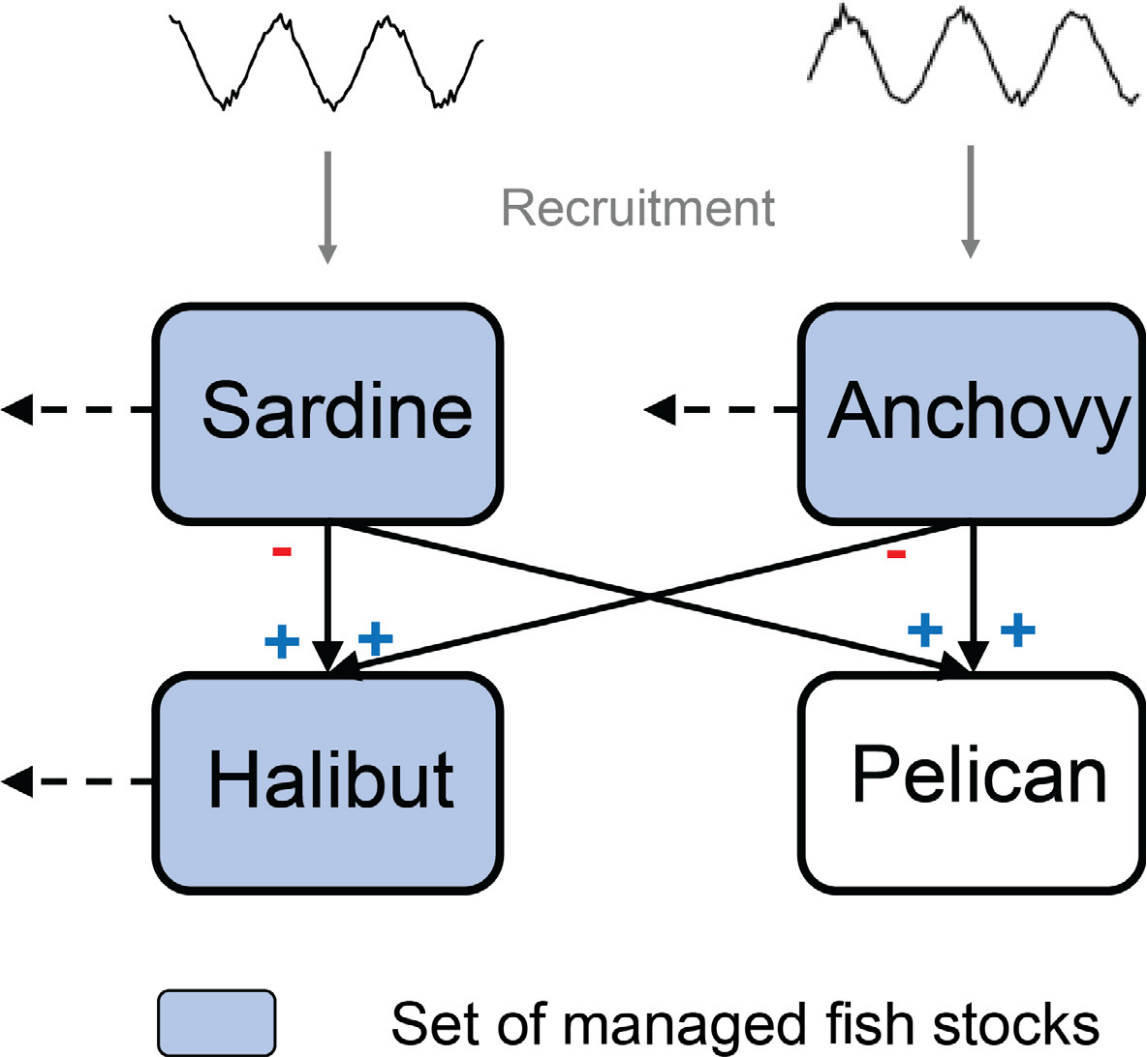
Ecosystem components and set of management regimes. Plus (minus) sign indicates linkage increases (decreases) population abundance. Solid lines represent ecological interactions and dashed lines represent fishing. We consider optimal single‐species management of each fish stock, optimal two‐species combinations, and optimal food‐web management under different assumptions of recruitment variability of the forage fish. We also consider the implications of constraints on Pelican stocks on economic and ecological outcomes of the fished species under the different management regimes.

To this end, we develop an economic‐ecological model for Pacific sardine (*Sardinops sagax*), northern anchovy (*Engraulis mordax*), and two generalist predators (see Fig. 1). One predator has a commercial value and is harvested (California halibut *Paralichthys californicus*) and the other predator (Brown Pelican *Pelecanus occidentalis*) is of conservation concern. California halibut are a high per unit value fishery managed by the California Department of Fish and Wildlife and over half of the landings are by bottom trawl (other gears include gill net and hook and line; Pomeroy et al. 2016). Brown Pelicans off of the west coast of United States were removed from the U.S. Endangered Species list in 2009 but recently the crash of sardine populations (Kuriyama et al. 2020) has led to concerns about their population status (Williams 2014).

With our coupled model, we ask the following questions: (1) How do the continuum of management regimes for the three harvested species from no management to single species to joint management impact the returns from fishing, ecological status of the fished stocks, and the non‐fished predator population (see Fig. 1)? (2) How do the answers to question (1) change under different assumptions regarding the environmentally driven productivity of the forage fish? (3) What are the economic and ecological implications when the non‐harvested population (Pelican) is brought into the management system as constraints that prohibit the non‐harvested population from going below a threshold level over time? And (4) how do the optimal fishery management regimes for sardines that we investigate in this paper compare across ecological and economic outcomes to stylized catch control rules that mimic current sardine management (NPFMC 2019) and a proposed harvest rule that adaptively lowers fishing rates when stock abundance is low (Essington et al. 2015*a*)?

While there are many existing modeling explorations into fishery and food web dynamics to support EBFM (see, e.g., Finnoff and Tschirhart 2003*a*, Fulton et al. 2011, Punt et al. 2016, Walters et al. 2016), relatively few have adopted an optimization framework (see, e.g., Wilen and Brown 1986, Clark 1990, Kellner et al. 2011, Essington et al. 2018). Here we aim to contribute to this body of knowledge by deriving optimal benchmarks that illuminate the trade‐offs of different management regimes. We also quantify the potential ancillary benefits from adopting EBFM on a key non‐fished species (Finnoff and Tschirhart 2003*b*) and the implications on fished species management outcomes when constraints are imposed to ensure that the non‐fished species does not go below a threshold. These latter constraints could arise, for example, from conservation measures resulting in controls on fishing, such as in the case on Alaskan fisheries and Steller sea lions (*Eumetopias jubatus*; NPFMC 2002) and forage fisheries and African Penguins (*Spheniscus demersus*; Sherley et al. 2018).

A key feature of forage fish populations is their cyclic dynamics, caused by fluctuations in recruitment (Lindegren et al. 2013, Szuwalski et al. 2019). This variability is important in at least two ways. One, harvest policies need to be responsive to rapidly changing productivity, which is often achieved through adaptive harvest control rules that reduce fishing mortality rates as populations decline (Pikitch 2015, Siple et al. 2020). Two, these fluctuations can impact dependent predators, even in the absence of fishing. The effect of these fluctuations on predators likely depends on whether fluctuations of species comprising the forage fish guild are in phase or out of phase with each other (Siple et al. 2020). When cycles are out of phase, the marginal effect of fishing one forage fish on predator's food availability is buffered by the availability of the second forage fish. However, when populations are in phase (synchronous), this buffering capacity is diminished and predator may be more sensitive to fishing on either species. For this reason, our model considers three different cases (no variability, synchronous variability, anti‐synchronous variability) to account for the range of observed covariance in anchovy and sardine dynamics globally (Siple et al. 2020) and to separate out the effects of population variability on optimal policies and outcomes.

Overall, we find significant gains in moving to integrated catch control rules both in terms of the economic gains of the fished stocks, and in terms of the impacts on the Brown Pelican populations. While several simulation studies investigate the use of ad hoc fishing moratoriums on forage fish when their populations go below a threshold (Smith et al. 2011, Pikitch et al. 2012), we find that moratoriums are part of an optimal fishing strategy that maximizes economic returns from the system. When we introduce constraints on the managed system due to conservation concerns, we find that optimal management anticipates that these constraints might bind in the future and acts early to avoid them. We also find that the additional degrees of freedom that EBFM approaches introduce for policymakers leads to the achievement of non‐fishery objectives at lowest costs to the fishing sectors. When comparing relative performance of current stylized catch control rules to optimal single species and optimal EBFM across ecological and economic dimensions, we find that the former trade‐off considerable economic value for ecological goals. For example, in the worst case scenario for Pelicans (when anchovy and sardine fluctuate synchronously), the two harvest control rules led to higher Pelican abundance than the optimal EBFM solution but the overall value of the fishery was on the order of 70% lower. These results highlight the potential space to negotiate win‐win solutions for forage fish management that are less conservative than the current and proposed catch control rule on the upswings of the population. While our base model assumed very tightly coupled dynamics between Pelicans and forage fish, our results are robust to a range of economic and ecological sensitivity analysis.

## Bioeconomic Model

We develop a stylized California Current ecological model of California halibut, northern anchovy, pacific sardine, and Brown Pelican population (see Fig. 1). We consider a continuum of management possibilities ranging from all fished species are not managed (open access), all fished species are closed to fishing, and single‐species optimal management to all four are optimally managed (Table 1). We investigate the implications of these fishery management regimes on the economic value of the system, ecological conditions of the fished species, and status of the Pelican stocks (see Fig. 1) under different assumptions regarding environmental variability (constant, synchronous, and asynchronous). In what follows, we describe components of the coupled economic‐ecological model. See supplementary information for details on the parameterization of the model components along with information on the numerical methods.

**Table 1:** Management regimes.

Label	Definition
H	Halibut optimal, sardine and anchovy open access
S	Sardine optimal, halibut and anchovy open access
A	Anchovy optimal, sardine and halibut open access
H + S	Halibut and sardine optimal, anchovy open access
H + A	Halibut and anchovy optimal, sardine open access
A + S	Sardine and anchovy optimal, halibut open access
A + S + H	Anchovy, sardine, and halibut optimal (full EBFM)

Note

In addition to these analyses, we also consider the case where all species are under open access (no management) and a permanent moratorium on fishing for all three species.

We emphasize at the outset that there is limited information on how vital rates of Brown Pelicans respond to anchovy and sardine abundance. We approached this uncertainty in two ways. One, our base model was purposefully parameterized to illustrate optimal harvest pathways and EBFM benefits under the most sensitive case, whereby both reproductive success and adult survivorship depend on prey availability. This choice allowed us to identify more clearly the types of benefits (and associated economic costs) that can be achieved in a particularly tightly linked food web. Two, we apply sensitivity analysis to see how much the findings depend on these strong linkages by systematically removing functional response relationships linking prey availability to Pelican vital rates.

### Ecological model

We discuss in turn the structure of the population dynamic model of the fished species that is adapted from Essington et al. (2018) and the Pelican population dynamics that is adapted from Punt et al. (2016).

#### Forage species

Consistent with empirical models of forage fish dynamics (Siple et al. 2020), we incorporate environmentally driven productivity of the forage fish. We also model population dynamics in numbers (*N_i_* (*t*)) and biomass (*X_i_* (*t*)) of the forage fish to account for variable recruitment driven by the environment, the primary mechanism by which populations and environments are linked (MacCall 2009). This also introduces time delays between juvenile stages of the population and age at recruitment into the fishable stock (τ*_i_* = *t *− η*_i_*, where η*_i_* is age at maturation and *t* denotes time). The state equations for two forage species are (*i* = a, anchovy, *i* = s, sardine, *i* = h, halibut)

(1) $$\frac{dX_{i}\left(t\right)}{\mathrm{dt}}\;=\;r_{i}\left(t\right)w_{i,r}\;+\;\kappa_{i}w_{i,\infty }N_{i}\left(t\right)+\left(M_{i}\;+\;P_{i}\left(X_{a}\left(t\right),X_{s}\left(t\right),X_{h}\left(t\right)\right)+F_{i}\left(t\right)\right)X_{i}\left(t\right)$$

(2) $$\frac{dN_{i}\left(t\right)}{\mathrm{dt}}\;=\;r_{i}\left(t\right)-\left(M_{i}\;+\;P_{i}\left(X_{a}\left(t\right),X_{s}\left(t\right),X_{h}\left(t\right)\right)+F_{i}\left(t\right)\right)N_{i}\left(t\right).$$

The components of Eqs. 1, 2 that capture the growth of biomass or numbers from one period to the next include *r_i_* (*t*), the time‐varying recruitment rate; *w_i_*_,_ *_r_*, the mass of new recruits; κ*_i_w_i_*_,∞_ *N_i_*, the growth rate of adults described by the asymptotic mass (*w_i_*_,∞_); and a metabolic rate parameter (κ*_i_*), as derived from a delay‐differential model (Quinn and Deriso 1999).

The time‐varying recruitment rates fluctuate according to the exponential of a sin function where

(3) $$r_{i}\left(t\right)={\bar{r}}_{i}e^{A_{i}\sin\left(\frac{2t\pi }{p_{i}}\;+\;\frac{s_{i}}{180}\;+\;\pi \right)\;-\;\frac{A_{i}^{2}}{4}}$$

where ${\bar{r}}_{i}$ is the average recruitment, *A_i_* is the amplitude, *p_i_* is the period, and *s_i_* is the starting position on the cycle. The last term is a bias correction to ensure that the mean of the recruitment time series equals ${\bar{r}}_{i}$, derived from the variance of a sine wave equaling $A_{i}^{2}/2$. We consider constant recruitment rates, synchronous, and anti‐synchronous rates between the forage species.

In Eqs. 1 and 2, the mortality rate in each period includes natural mortality (*M_i_*), predation that are a Type‐II function of the forage species and halibut (*P_i_* (*X*_a_, *X*_s_, *X*_h_)), and fishing morality that varies in time *t* (*F_i_* (*t*)). Predation rates are calculated from the predator functional response as described in Essington et al. (2015*a*):

(4) $$P_{i}\left(∙\right)\;=\;\frac{C_{\max}\alpha_{i}X_{h}\left(t\right)}{C_{\max}\;+\;\alpha_{i}X_{i}\left(t\right)\;+\;\alpha_{j}X_{j}\left(t\right)\;+\;Y}$$

where *C*_max_ is the maximum specific consumption rate of the predator, α*_i_* and α*_j_* are the effective search and capture rate on prey *i* and *j*, *Y* is consumption of other prey by the predator. Following Punt et al. (2016), we assume predation by Brown Pelicans on forage stocks is minimal compared to other mortality sources (<3% of total mortality; Koehn et al. 2017).

#### California halibut

We also model population dynamics in numbers (*N_i_*) and biomass (*X_i_*) of the California halibut to account for time delays (τ*_i_*) between juvenile stages of the population and age at recruitment into the fishable stock. The state equations for California halibut are (*i* = a, anchovy; *i* = s, sardine; *i* = h, halibut)

(5) $$\frac{dX_{i}\left(t\right)}{\mathrm{dt}}\;=\;R\left(X_{i}\left(\tau_{i}\right)\right)w_{i,r}\;+\;\kappa_{i}w_{i,\infty }\left(X_{a},X_{s}\right)N_{i}\;+\left(M_{i}\;+\;\kappa_{i}+F_{i}\left(t\right)\right)X_{i}$$

(6) $$\frac{dN_{i}\left(t\right)}{\mathrm{dt}}\;=\;R\left(X_{i}\left(\tau_{i}\right)\right)\;-\;\left(M_{i}\;+\;\kappa_{i}\;+\;F_{i}\left(t\right)\right)N_{i}$$

where *R*(*X*_h_(τ*_i_*)) is the recruitment rate, *w_i_*_,∞_(*X*_a_, *X*_s_) is the asymptotic mass gain, and all other parameters are defined as in Eq. 1. Following Essington et al. (2001) and Essington et al. (2018), the asympototic weight gain function is responsive to changes in the halibut consumption rate of the forage species by applying an energetic‐based model (generalized von Bertalanffy) that relates consumption to growth (details are provided in Essington et al. [2015*a*]). Recruitment follows a Beverton‐Holt relationship with

(7) $$R\left(X_{h}\left(\tau_{i}\right)\right)=\frac{aX_{h}\left(\tau_{i}\right)}{1+bX_{h}\left(\tau_{i}\right)}e^{‐M_{j}\tau_{i}}$$

where *X*_h_(τ*_i_*) is the lagged value of halibut biomass, *a* and *b* are the parameters of the functional response, and *M_j_* is the density‐independent mortality in the stages prior to recruitment. Parameters of the Beverton‐Holt were fit to estimated stock biomass and recruitment (Maunder et al. 2011)

#### Brown Pelican

We modeled numbers of Brown Pelicans (subscript bp) as a function of reproduction and survivorship, largely following the model structure detailed by Punt et al. (2016). The population dynamics in numbers are

(8) $$\frac{dN_{\text{bp}}\left(t\right)}{\mathrm{dt}}\;=\;R_{\text{bp}}\left(X_{a}\left(\tau_{a}\right),X_{s}\left(\tau_{s}\right),N_{\text{bp}}\left(\tau_{\text{bp}}\right)\right)-M_{\text{bp}}\left(X_{a}\left(t\right),X_{s}\left(t\right)\right)N_{\text{bp}}\left(t\right)$$

where *R*_bp_(*X*_a_(τ_a_), *X*_s_(τ_s_), *N*_bp_(τ_bp_)) is recruitment depending on the lagged values of forage fish abundance and Brown Pelican numbers, and *M*_bp_(*X*_a_(t), *X*_s_(t)) is the mortality rate depending on the current values of forage fish abundance. The model allows for reproductive success and subsequent survivorship to depend on forage fish abundance, expressed as a percent depletion from a baseline condition. Depletion at time *t* is additively separable in forage fish biomass density and is equal to

(9) $$d\left(X_{a}\left(t\right),X_{s}\left(t\right)\right)\;=\;\Gamma_{a}\frac{X_{a}\left(t\right)}{X_{\text{ao}}}+\Gamma_{s}\frac{X_{s}\left(t\right)}{X_{\text{so}}}+\Gamma_{\text{other}}$$

where Γ*_i_* is the fraction of diet at the baseline condition comprised of species *i*, *X_i_*_o_ is the long‐run average forage fish biomass in the absence of all fishing, and “other” reflects feeding on other prey whose abundance we assume is constant.

The number of successfully fledged chicks (reproduction) in time *t* is given by a density dependent function and by lagged prey availability

(10) $$R_{\text{bp}}\left(∙\right)\;=\;0.5e^{\left(-4{\bar{M}}_{\text{bp}\tau }\right)}N_{\text{bp}}\left(\tau_{\text{bp}}\right)\phi_{r}\left(t\right)\left(1+\left(\Phi -1\right)\left(1-{\frac{N_{\text{bp}}\left(\tau_{i}\right)}{K_{\text{bp}}}}^{z}\right)\right)$$

where Φ governs the intensity of density dependence, and *K*_bp_ is the Pelican abundance at which all females fledge 1 chick annually, and *z* is set to make net productivity greatest at 60% of *K*_bp_. The multiplication by 0.5 in Eq. 10 is introduced because we model the female portion of the population, and ${\bar{M}}_{\text{bp}\tau }$ is the (fixed) mortality rate in the years before age at maturation. This assumes that survivorship in this stage is constant based on the initial prey abundance. We assumed that this simplifying assumption did not have substantive effects on simulated Brown Pelican population dynamics. ϕ*_r_* (*t*) is a scalar that reduces reproduction based on prey abundance

(11) $$\phi_{r}\left(t\right)=\frac{\max\left[0,\left(1-\theta_{1}-\theta_{2}\right)\theta_{3}\left(d\left(X_{a}\left(\tau_{i}\right),X_{s}\left(\tau_{i}\right)\right)-\theta_{1}\right)\right]}{\left(1-\theta_{1}\right)\theta_{2}\left(1-\theta_{3}\right)+\left(\theta_{3}\left(1-\theta_{1}\right)-\theta_{2}\right)\left(d\left(X_{a}\left(\tau_{i}\right),X_{s}\left(\tau_{i}\right)\right)-\theta_{1}\right)}$$

where θ_1_ is the largest value of $d\left(∙\right)$ for which reproductive success is zero, and θ_2_ and θ_3_ govern the steepness of the function. The model functional forms and parameter values follow Punt et al. (2016) and parameter values are provided in Appendix S1: Table S2.

To model the mortality of adults that is a function of the forage species $M_{\text{bp}}\left(∙\right)$ in Eq. 8 we utilize the same functional form as Eq. 11. A key difference, however, is that survivorship depends on forage species biomass levels in *t*. Specifically, we have

(12) $$M_{\text{bp}}\left(X_{a}\left(t\right),X_{s}\left(t\right)\right)={\bar{M}}_{\text{bp}}-\log\left(\phi_{v}\left(t\right)\right)$$

where ${\bar{M}}_{\text{bp}}$ is the mortality rate when prey are abundant and do not reduce survivorship, and ϕ*_v_* (*t*) is equal to

(13) $$\phi_{v}\left(t\right)=\frac{\max[0,\left(1-\theta_{s1}-\theta_{s2}\right)\theta_{s3}\left(d\left(X_{a}\left(t\right),X_{s}\left(t\right)\right)-\theta_{s1}\right]}{\left(1-\theta_{s1}\right)\theta_{s2}\left(1-\theta_{s3}\right)+\left(\theta_{s3}\left(1-\theta_{s1}\right)-\theta_{s2}\right)\left(d\left(X_{a}\left(t\right),X_{s}\left(t\right)\right)-\theta_{s1}\right)}$$

where θ*_vi_* represent the effect of prey on survivorship.

Because there are no comprehensive, ecosystem‐wide estimates of Brown Pelican abundance (but see Koehn et al. 2017 for an estimate of biomass), we instead modeled relative changes in Brown Pelican population numbers. We set the initial number to 100 to reflect 100% of current levels. This relative measure of Brown Pelican population allowed for easier comparison of the relative effects of different management measures.

### Economic model

We choose to represent the economic portion of the coupled ecological‐economic model with a simple structure of fishing profits while still providing a realistic description of fishery systems (Clark 1990). Total profits for each fishery (sardine, anchovy, and halibut) are quadratic in their own fishing effort, which approximates more complicated relationships, and are the difference between catch value (total revenue) and the fishing effort costs required to obtain that level of catch.

In each period, the economic value of fishing for each fished species is

(14) $$\Psi_{i}\left(X_{i}\left(t\right),E_{i}\left(t\right)\right)\equiv \left({\tilde{p}}_{i}q_{i}X_{i}\left(t\right)-\left[c_{i1}+c_{i2}E_{i}\left(t\right)\right]\right)E_{i}\left(t\right)$$

where I(*t*) depends on the landings price ${\tilde{p}}_{i}$, the current biomass level of species *i*, and costs of fishing effort for species *i* that are sensitive to short‐run adjustment of fishing effort (via cost‐of‐effort parameters *c_i_*_1_ and *c_i_*_2_). The product of fishing effort and the catchability coefficient for the species (*q_i_*) yields the fishing morality rates *F_i_* (*t*) in the population dynamics.

### Management model

We assume a regulator that is knowledgeable and understands population dynamics and whose objective is to maximize fishing profits by choosing a path of fishing effort over time (*E_i_* (*t*)). The regulator maximizes the total net present value of the current and future profits of the species within the management system. When all of the species are optimally managed, the regulator solves the following problem

(15) $$J\left(E_{a}^{*}\left(t\right),E_{s}^{*}\left(t\right),E_{h}^{*}\left(t\right)\right)=\overset{\max}{E_{a}\left(t\right),E_{s}\left(t\right),E_{h}\left(t\right)}\int_{0}^{\infty }e^{-\delta t}\left[\sum_{i=[a,s,h]}\Psi_{i}\left(x_{i}\left(t\right)E_{t}\left(t\right)\right)\right]dt$$

subject to the population dynamics (Eqs. 1, 2, 5, 6, 8), non‐negativity conditions on the fish stocks and effort levels (see Appendix S1: Table S1), and initial conditions for the stock biomass and numbers (see Appendix S1: Table S1). δ is a conventional discount rate and $J\left(∙\right)$ denotes the net present value of the system evaluated at the optimal solution (denoted by *).

Choosing the effort levels for species *i* in period *t* to maximize Eq. 15 corresponds to the case where all three fished species are integrated in EBFM. Based on earlier studies, we know that as the quadratic cost term approaches zero, the optimal solution will be represented by a most rapid approach path to the singular solution (Spence and Starrett 1975). Given the variability in recruitment in the forage fish, the singular solution will not be constant over time (see, e.g., Stefanou and Wilen 1992). While the assumption of an omniscient regulator is obviously stylistic, these analysis are useful in understanding the mechanisms behind trade‐offs in complex systems.

In the cases where one or more species are outside of the management systems, also known as operating as open‐access fisheries, the level of fishing effort in each time period in those species *i* is determined by setting average profits (Ψ*_i_* (*X_i_* (*t*), *E_i_* (*t*))/*E_i_* (*t*)) to zero and the objective of the regulator is to optimally choose the effort levels in each *t* for the managed species. This assumes that open‐access fishing effort instantaneously adjusts to changes in fish biomass levels in each *t*. If, on the other hand, open‐access effort responds sluggishly to changes in average profits over time, we would expect higher peaks in abundance and potentially higher fishing rates at lower population levels. We follow the approach in Kellner et al. (2011) and Essington et al. (2018) for tractability of the numerical analysis and because similar assumptions underlie the ad hoc rules being applied to forage fish management.

## Results

We consider the implications of a continuum of management‐variability regimes (Table 1). The three scenarios on the time‐varying growth rates of anchovy and sardine populations are constant growth, synchronous growth where the anchovy and sardine stocks are moving in tandem with the same amplitude and wave length, and anti‐synchronous growth where the anchovy and sardine stocks are moving in opposition to each other with the same amplitude but opposite cycle. The worst case scenario for Pelicans (seabirds more generally) is synchronous growth due to the simultaneous lows in abundance in their prey populations.

In the sensitivity analysis, we illustrate the case where there is both anti‐synchronous variability and sardines have a longer frequency. We also include robustness checks in terms of higher discount rate (10%), higher prices per unit mass for the forage species (25%), and where initial stock levels are 50% of the base case initial conditions. Because our base model formulation captures the case where the Pelican stocks are highly dependent on the forage fish (because adult survivorship declines with decreasing prey abundance), we also check the robustness of our results to cases where either adult Pelican survivorship is independent of the forage stocks (θ_2_ *_s_* = 0 in Eq. 13) or where reproductive success of Pelicans is independent (θ_2_ = 0 in Eq. 11). Both of these formulations decouple the dynamics of Pelicans from the forage fish dynamics to an extent.

For each management regime variability pairing, we illustrate the biomass levels, fishing effort levels, and the impacts on the Pelican populations over time. We also calculate the net present value of the different fishery management regimes.

### Dynamics of fishing regimes

**Fig. 2:**
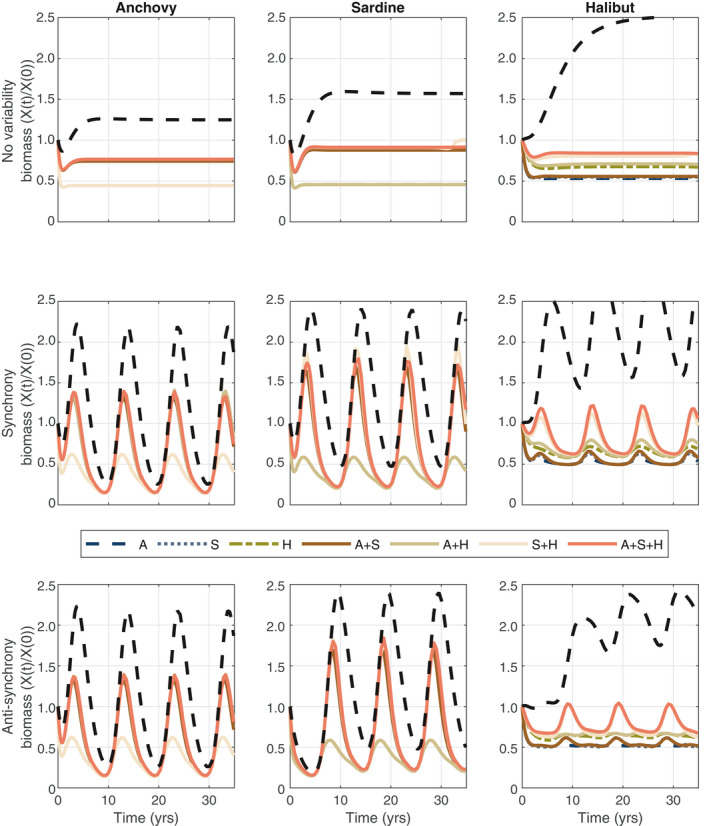
Biomass over time (years) for the management regime–variability pairs. Biomass is scaled off of the initial conditions to facilitate comparisons across the panels (*X*(*t*)/*X*(0)). Management regime labels are found in Table 1. The top row corresponds to no variability, middle row to synchronous variability, and the bottom row to anti‐synchronous variability. The first column is anchovy, second column is sardine, and third column is halibut. The black dashed line is the population dynamics with no fishing for any species.

In the case of no variability (top row of Fig. 2), there are two scenarios for the forage species that provide quantitative differences: either the species are under optimal management or they are not under management (open access). Jointly optimal outcomes for forage fish are virtually the same, implying that forage fish management is not sensitive to management of other species in the system. This might seem counterintuitive when it comes to predator–prey systems, but it follows from the relatively weak top‐down control that halibut exert on sardine and anchovy in this system. In other model systems, the prey species are sensitive to how the predator populations are managed (see, for example, Kellner et al. 2011, Essington et al. 2018). On the other hand, halibut exhibit different optimal dynamics depending on the management regime, where the highest biomass levels are under full EBFM. Overall, halibut biomass levels are higher over time when its management is paired with one of its prey species. Not surprisingly, for each species the lowest population levels are when it falls outside the management system under open‐access conditions.

Introducing growth variability naturally leads to cycles in the optimal biomass dynamics, but the overall ordering of the biomass levels over time are qualitatively similar to the case of no variability. However, optimal fishing does impact both the timing and magnitude of the peaks and troughs of the cycles relative to the case of open access and no fishing. For example, when sardines are optimally managed, their peak is slightly later and much higher than when they are under open access but both peaks and troughs are earlier and lower than when no fishing is occurring (black dashed line in Fig. 2). This finding is consistent with Essington et al. (2015*b*). Halibut absorbs the variability in growth in its prey species experiencing different waves depending on the management regime.

The fishing mortality rates across the management‐variability pairs are unsurprisingly highest under open‐access levels (see Appendix S1: Fig. S1). In the presence of the growth variability, optimal fishing pathways include fishing moratoriums; these stop‐gap measures on fishing mortality are driven by the economics of the problem rather than imposed on an ad hoc basis (see Pikitch 2015) for a discussion on how stop‐gap measures should be imposed on forage fish stocks to protect their predators and to buffer against uncertainty). Interestingly, moratoriums are found both under open access (it is no longer profitable to fish) and in the optimal fishing mortality solutions, where the latter are longer in duration and on a different schedule over time. The timing of the moratoriums is such that they are in place before the stocks reach their lowest point in their cycle. That is, the switch is made to stop fishing in anticipation of the lowest point in the population cycle and fishing does not resume until the population is increasing again. We also find that halibut experience fishing moratorium as the variability in their prey ripples through the system. These patterns of fishing mortality, including the moratorium, are optimal for maximizing the economic returns to the fishery.

While the solution of Eq. 15 for the full‐EBFM case (A + S + H) is the optimal set of fishing mortality rates over time for each species to maximize the joint returns, an integrated approach results in trade‐offs at the stock level in our system. For example, the net present value from anchovy is higher when it is decoupled from the management of sardine and halibut (see Appendix S1: Fig. S2). On the other hand, halibut performs much worse when its prey species are not part of the management regime (see Appendix S1: Fig. S2). Sardine exhibit similar trade‐offs as anchovy except for the case when sardine is paired with halibut. In this case, the magnitude of the trade‐off is negative or small. The patterns in the trade‐offs are robust to the nature of the variability as are the levels.

### Impacts of fishing regime on Pelican population

**Fig. 3:**
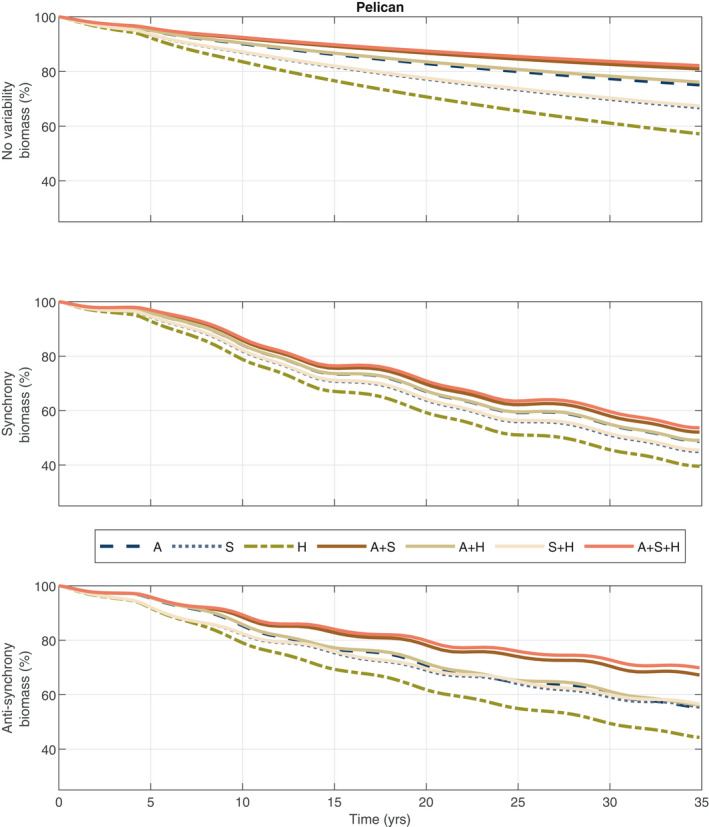
Pelican numbers over time (years) under the management–variability pairs. Management regime labels are found in Table 1. The top row corresponds to no variability, middle row to synchronous variability, and the bottom row to anti‐synchronous variability. Pelican numbers measure percentage of the level at time 0.

We find that the Pelican stocks levels are highest under ecosystem‐based fishery management over time and worse off when their prey species are both under open access (A + S + H vs. H regimes in Fig. 3). The largest decline in the population over time is under synchronous growth, where both prey species experience lows at the same time. Some have argued that forage fish biomass should remain above 40% unfished biomass levels to support the dynamics of predators, such as Pelicans. In Appendix S1: Fig. S3, we add the 40% threshold at the individual species level for sardine and anchovy to Fig. 2. At our base case, we find that the population dynamics when the forage fish are optimally managed have higher high populations and lows similar to but lower than the 40% threshold.

Across all of the management‐variability pairs, a natural question to ask is, what are the possible long‐run gains in Pelican populations from adopting different management measures? To answer this question, we investigate the long‐run gains for each management regime against the case where all fisheries are open access. We also consider the case where a permanent moratorium is placed on the managed fisheries. The latter case illustrates the greatest gains possible for the Pelican population given the species under management control and provides another benchmark for optimal fishing rates. For example, we consider the case where anchovy and sardine are optimally managed against the case where all three species are open access and the case where anchovy and sardine are not fished but halibut is under open access.

**Fig. 4:**
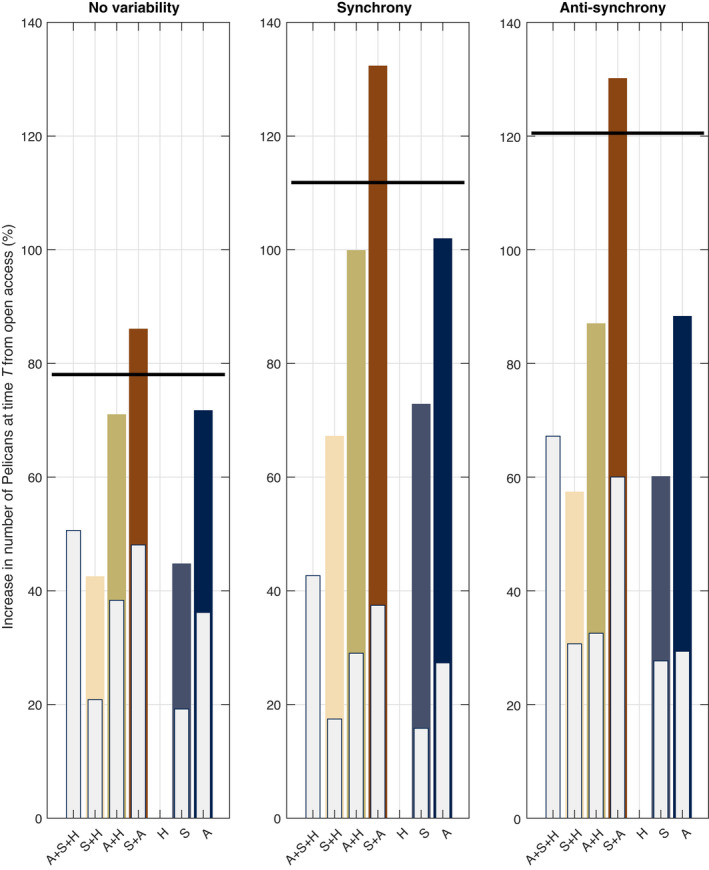
Long‐run Pelican numbers under the management–variability pairs. The white bars measure the increase by bringing species into the optimal management regime case relative to the case where all fisheries are open access. For example, the ecosystem‐based fisheries management model (EBFM) illustrates a 30% increase in Pelican stocks relative to the case of open access. The dark bars represent the highest possible achievement if the fisheries under management were under a fishing moratorium. For example, the dark bar in S corresponds to the case where A and H are open access and the sardine fishery is closed to fishing. The black line corresponds to the percentage gains in the long‐run average Pelican stock when *all* species are not fished.

We find that in general bringing species into the management regime (white bars) will improve Pelican long‐run numbers from when they are fished at open‐access levels, except for halibut where optimally managing halibut only is no different for long‐run Pelican numbers. We also see that the greatest gains occur either under full EBFM or when S + A are optimally managed. Not surprisingly, creating moratoriums for the forage species (dark bars) leads to large gains but of course this is at the cost of not being able to fish those species. We also find that a fishing moratorium on the forage species along with halibut operating under open‐access (and therefore at lower biomass levels) leads to a higher long‐run Pelican population than the long‐run solution with no fishing on all three species (black bar in Fig. 4). This is because open‐access fishing for halibut slightly releases the forage fish from halibut predation resulting in higher numbers of forage fish than otherwise would be the case if halibut was not fished.

Fig. 4 highlights the potential gains in each management regime in the long‐run from open access and also illustrates the maximum possible gains if no fishing occurred on the management stocks. We ask next, what are the costs from imposing a constraint that Pelican populations cannot drop below at any time? The threshold level is specific to each management–variability pair, where we calculate the maximum possible gain between optimal fishing (height of white bar in Fig. 4 and $N_{\text{bp}}^{*}\left(T\right)$ in Eq. 16) and no fishing (height of dark bar in Fig. 4 and *N*_bp_(*T*)|*_f_* _= 0_ in Eq. 16) and then constrain the management regime to not allow Pelican stocks in any time period to fall below 5–20% of this gain (ψ in Eq. 16 ranges between 0.05 and 0.2). We then measure the loss in net present value (NPV) from the unconstrained case ($J^{*}\left(∙\right)$ in Eq. 15) against the situation where we solve Eq. 15 with Eq. 16 imposed.

(16) $$N_{\text{bp},i}\left(t\right)\geq \psi *\left(N_{\text{bp},i}\left(T\right){|}_{f=0}‐N_{\text{bp},i}^{*}\left(T\right)\right)+N_{\text{bp},i}^{*}\left(T\right)$$

where *i* is the management regime.

Intuitively, the constraint on Pelican numbers will not be binding in the early years of management, but because the manager foresees that the constraint is going to bind in the future, they begin to change fishing mortality rates sooner to avoid crossing the threshold. For example, when Ψ = 0.2, the optimal solution without the constraint crosses the threshold imposed by Eq. 16 around 30 yr (see *T*_cross_ in Appendix S1: Table S5). With the constraint imposed, however, the manager begins to adjust their actions (changes in fishing mortality rates) long in advance of the time at which the constraint would bind. At the base case parameters, these changes begin to occur around 5 yr (see *T*_change_ in Appendix S1: Table S5). These anticipatory changes in fishing mortality are driven by the fact that adjusting the stocks of halibut, anchovy, and sardine takes time due to the growth characteristics of the stocks and that there are adjustment costs to changing fishing mortality, both of which act to smooth out changes over time.

Another benefit of EBFM and one that the literature has not highlighted is in the degrees of freedom within which policy goals can be accomplished. For example, in the case where all species are under optimal management, the degrees of freedom available to the manager to meet the constraint is the greatest. Specifically, we find that fishing mortality rates are generally lower for the forage stocks (up to 20%) and higher for halibut (<10%) with the constraint than without it. When only one fishery is under optimal management, the manager is severely constrained in the ways in which it can ensure to meet the constraint (only adjust the fishing mortality of the one species under management). The extreme example of this in our system is halibut. Here the regulator cannot meet the Pelican constraint by only managing halibut even if the fishery is shut down or if it is fished at open‐access levels, as anchovy and sardine are outside the management system and under open‐access conditions (see row H in Appendix S1: Table S5). This result stems from the strength of the coupling (or lack thereof) between halibut and the forage stocks. In other systems, it might be possible to fish halibut at high enough rates to release the prey (here forage stocks) from predation that in turn would help the Pelican stocks (or more generally other predators). Another example of the degrees of freedom is the delay in imposing changes to meet the constraint when all of the stocks are optimally managed (see *T*_change_ in the A + S + H row in Appendix S1: Table S5).

We also calculate the elasticity of net present value with an increase in Pelican years (ɛ_NPV/P_), which provides a unitless measure of the responsiveness in the cost of meeting the constraint (reduction in net present value) with an increase in the total Pelican years. Specifically, the elasticity is equal to the ratio of the percentage change in net present value and Pelican numbers

(17) $$\epsilon_{\text{NPV/P}}=\frac{J^{*}{\left(∙\right)}_{\Psi \;=\;0}-J^{*}{\left(∙\right)}_{\Psi }}{J^{*}{\left(∙\right)}_{\Psi \;=\;0}}/\frac{{\text{TN}}_{\text{bp},\Psi \;=\;0}-{\text{TN}}_{\text{bp},\Psi }}{{\text{TN}}_{\text{bp},\Psi \;=\;0}}$$

where $J^{*}\left(∙\right)$ is the net present value in the unconstrained (Ψ = 0) and constrained cases (see Eq. 15) across all management–variability pairs, and TN_bp_ is the (non‐discounted) sum over the horizon length of Pelican numbers in the two cases. This latter term is equivalent to the concept of Pelican life‐years where the higher the total, the larger the cumulative total of Pelicans there are throughout the time horizon. An elasticity greater than one corresponds to a 1% increase in the Pelican numbers leading to a larger than 1% decrease in the net present value. We find, across all variability scenarios, that optimally managing sardine only has the largest elasticity and greater than one. This is not surprising given the importance of sardine in the diet of the Pelicans and that there are limited ways to meet the constraint other than reducing the fishing mortality on the sardine stocks, which leads to large reductions in net present value. Consistent with the long‐run results in Fig. 4, we find the lowest elasticity when anchovy and sardine are optimally managed together (and halibut open access), where a 1% increase in Pelican numbers corresponds to a <1% drop in net present value. We also find an elasticity less than one when all three species are optimally managed together (A + S + H row in Appendix S1:Table S5). This result highlights how EBFM can be a mechanism to address conservation constraints on fishery management at the lowest possible cost in aggregate terms. However, as the net present value analysis demonstrates, there are still underlying trade‐offs across the fisheries.

### Comparing EBFM and catch control rules for sardines

Across the management–variability pairs, we solved for the optimal set of fishing moralities for each species over time. Here we investigate how the optimal harvest control rules for sardines compare to those advocated in the literature (e.g., Siple et al., 2019) and utilized in practice. We approximate the current harvest guideline (HCR) for sardines as

(18) $$\begin{array}{ll} & C_{\text{HCR}}\left(t\right)=\\ & \left(\begin{array}{cc} 0 & \text{if}\;X_{s}\left(t\right)\leq 150,\;000\\ C_{\text{bar}}=0.87\left(X_{s}\left(t\right)-150,\;000\right)F_{s}\left(t\right) & \\ 200,\;000 & \text{if}\;C_{\text{bar}}\geq 200,\;000 \end{array}\right. \end{array}$$

where 0.87 corrects for the fish in Mexico and Canada, and *F*_s_(*t*) = (0.0465824 + 0.06224328 $r_{s}\left(t\right)/{\bar{r}}_{s}$) is the fishing exploitation fraction, which is a function of the recruitment deviation due to the variability in the system. Following Siple et al (2019), we employ a basic hockey stick rule that mimics the rule advocated for in Pikitch et al. (2012). The formulation is

(19) $$C_{\text{HS}}\left(t\right)=\left(\begin{array}{cc} 0 & \mathrm{if}\;X_{s}\left(t\right)\leq 0.4X_{s}o\\ \frac{0.5}{0.4}M_{s}\left(\frac{X_{s}\left(t\right)}{X_{s}o}-0.5M_{s}\right) & \\ 0.5M_{s} & \text{if}\;X_{s}\left(t\right)\geq 0.8X_{s}o \end{array}\right.$$

where *M*_s_ is the natural mortality of sardines and *X*_s_ *o* is the unexploited level of sardines (long‐run average over 200 yr).

**Fig. 5:**
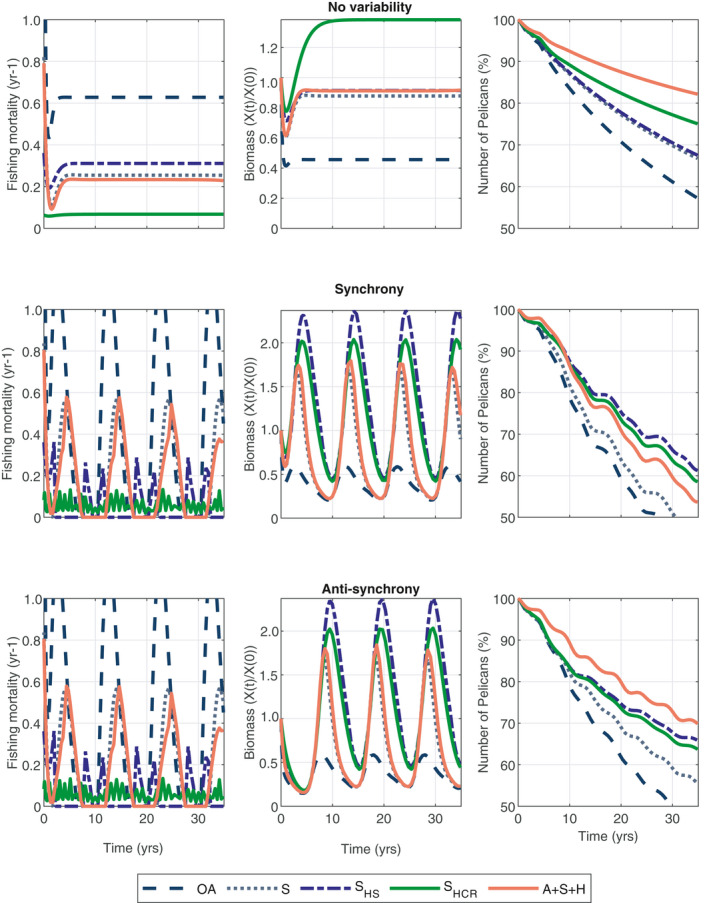
Sardine management (fishing mortality rates and biomass) and Pelican numbers over time. The legend is as follows: OA corresponds to open‐access, S corresponds to optimal management of sardine only, S_HS_ is the hockey‐stick formulation for the catch control rule, S_HCR_ is the harvest control rule, and A + S + H is the full‐EBFM optimal. The first column are fishing mortality rates (yr‐1), second column is scaled biomass levels (*X*(*t*)/*X*(0)), and the third column are Pelican numbers over time (% off of initial level). The top row is the no variability case, middle row is synchronous, and bottom row is anti‐synchronous.

We compare these two catch rules with the open‐access levels, sardine managed optimally, and when sardines are managed optimally in the full‐EBFM solution (see Fig. 5). A number of observations are noteworthy. First, the time series of exploitation rates are considerably different between the optimal solution and those generated by applying harvest control rules (Fig. 5). Specifically, the optimal solution involves much more variable exploitation rates, where rates increase to high levels (between 0.4 and 0.6) during high productivity regimes, followed by extended periods lasting roughly 5 yr of no fishing during lower productivity regimes. In contrast, the generic hockey stick control rule and the current harvest control rule produce smaller fluctuations in exploitation rate that are generally far lower than the peaks produced in the optimal case, and involve fewer fishery closures. Second, both of the harvest control rules reduce the NPV of the sardine fishery by considerable amounts, ranging from 60% to 80% loss of NPV (Appendix S1: Table S6). Thus, the harvest control rules enhance stability at the cost of maximizing net present value. Third, and most importantly, the harvest control rules appear to provide comparable conservation benefits to seabirds as the full EBFM optimal solution (Fig. 5). In the worst case scenario for seabirds (when anchovy and sardine fluctuate synchronously), the two harvest control rules led to higher Pelican abundance than the optimal solution that considered net present value of anchovies, sardines, and halibut together. The two harvest control rules resulted in slightly lower Pelican densities than the EBFM‐optimal solution when sardine and anchovy fluctuated anti‐synchronously, but Pelican outcomes from applying harvest control rules were still far better than the single‐species optimal solution or the open access outcome (Fig. 5).

### Sensitivity analysis

To check the robustness of our results, we ran a number of sensitivity analysis, including higher discount rate (10%), higher prices per unit mass for the forage species (25%), and cases where the adult survivorship of Pelicans is independent of the forage stocks (θ_2_ *_s_* = 0 in Eq. 13), where recruitment of Pelicans is independent (θ_2_ = 0 in Eq. 11), and where the initial stock levels are 50% lower than in the base.

We find, across all of these analyses, that the qualitative results with regard to economic rankings of the different management regimes and impacts of the management regimes on the Pelican stocks are robust (see Appendix S1 for full set of results). Slight differences do exist, however. For example, with a higher discount rate that puts more weight on getting economic returns in the present, the peaks in the cycles of sardine and anchovy when they are optimally fished are sooner in time relative to the unfished cycles (Appendix S1: Fig. S6). Decoupling survivorship or recruitment reduces the dependency of Pelicans on the forage fish and therefore, the Pelican numbers are less impacted by fishing of the forage fish, everything else being equal (see, e.g., Appendix S1: Fig. S10 and Appendix S1: Fig. S12). The decoupling is more pronounced in the case of survivorship. When the initial stock levels are lower, the optimal fishing policies are to rebuild the stocks, which, in this case, introduces a moratorium on halibut fishing (Appendix S1: Figs. S14, S15).

One significant qualitative change occurs when the anti‐synchronous variability is driven by sardines having longer frequency recruitment fluctuations (see Appendix S1: Fig. S4). In this case, we find that the hockey‐stick outperforms EBFM in the long‐run in terms of minimizing the impact on the Pelican stocks. The differences between EBFM, the hockey‐stock and catch control rule are not very large, however, and the ordering varies over time (Appendix S1: Fig. S5).

## Discussion

Forage fish play a special role in marine food webs while also being important sources of protein for human consumption either directly or indirectly as feed into livestock and aquaculture operations. Consequently, calls to reduce fishing pressure on forage fish globally (Smith et al. 2011) and in particular with Pacific sardines (Shively 2015) are often countered with claims about their importance to food provision and security in many parts of the world (Alder et al. 2008). One complicating factor in managing forage fish is the environmentally driven variability in recruitment, which can mask the underlying health of the stock and impacts of fishing and require management that can adapt to rapidly changing productivity. Here we developed a coupled ecological‐economic model that accounts for environmentally driven variability of recruitment to better understand the optimal benefits of ecosystem‐based management on fished stocks and ancillary benefits on Pelicans (or unfished stocks).

While the empirical literature is mixed on the ability to detect a signal of fishing on forage fish stocks and their predators (Essington et al. 2015*b*, Hilborn et al. 2017, Pikitch et al. 2018), our model results demonstrate (maybe not surprisingly) a tight coupling between fishing effort and population dynamics of the forage fish. Specifically, fishing combined with the management regime changed the amplitude and wavelength of the stocks. Interestingly, we find optimal fishing moratoriums occurring at forage fish population thresholds greater than those advocated for in the literature to trigger fishing moratorium. Moratorium could, however, have significant displacement costs on fishing dependent communities due to the loss of catch and employment. While we did not take those costs into account in this paper, future research could investigate the impacts of these costs on the optimal fishing patterns either through the inclusion of a constraint that requires some minimum fishing effort level or an additional cost in the objective function. We also considered the case where the regulator is able to adjust management on the relevant time scales. Another formulation could consider the role of policy adjustment costs (see, e.g., Ryan et al. 2017).

At the same time, our optimal EBFM solutions extract the greatest overall value from the system over time, levels significantly larger than those derived from ad hoc catch control rules. This greater value derives from higher fishing mortality rates when the populations are near the crest of their cycles. Still the EBFM gains are not evenly distributed across the species (see also Essington et al. 2018), highlighting that some sectors (species and gear combinations) might be more favorable to applying EBFM approaches than others.

Another benefit of integrated management of fisheries, such as under EBFM, is the additional degrees of freedom that regulators have in meeting non‐fishery goals. We showed, for example, that when halibut are the only managed stock that the regulator was not able to keep the Pelican population above the threshold levels. Overall, we found that the more management levers there are for decision‐makers, the more likely the non‐fishery goals (here constraints on the Pelican population) can be meet at the lowest possible cost.

While it is certainly possible that ad hoc catch control rules that are more conservative than the optimal rules we derived can outperform on non‐fishery objectives, we found that fully integrated management was almost as good on the non‐fishery objectives even though those objectives were not part of the optimization. Overall, our results highlight that there is potential space to negotiate win‐win solutions for forage fish management that are less conservative on the upswings of the population. But these solutions require taking an ecosystem‐based fishery management perspective and quality information on stock levels.

Our model necessarily made several simplifications. For example, we did not account for stochasticity, where shocks could lead to populations crossing thresholds in unexpected ways (see, e.g., Donovan et al. [2019] for analysis that incorporates thresholds into stochastic resource management). We assumed that fisheries not managed were characterized by open‐access effort levels adjusting instantaneously. We expect that sluggishness of fishing effort in the entry and exit decisions will impact the amplitude and duration of the population cycles and could lead to higher fishing rates than we found during periods of low population abundance. We also do not consider management uncertainty stemming from implementation errors or errors in inaccurate assessments of the stocks (see, e.g., Memarzadeh et al. 2019). Indeed, this uncertainty (combined with potentially rapid changes in productivity) is one reason why “hockey ‐ stick” control rules can lead to improved fishery outcomes (Siple et al. 2019), because they are less likely to inadvertently overfish during periods of productivity decline (Essington et al. 2015*b*). While these shortcomings would need to be addressed in the development of tactical economic‐ecological models for management prescriptions, we hypothesize that the qualitative results regarding EBFM (economic values and degrees of freedom to meet non‐fishery goals at lowest possible (expected) costs) are robust to including these factors. More tactical ecological‐economic models could also be used to design and evaluate mechanisms that could be used to address the inequities that integrated (EBFM) management might yield in any one fishery.

## Supporting information

Appendix S1

## Data Availability

Sample matlab code can be found at https://doi.org/10.5281/zenodo.4660540.
