# Supplementary material for: Direct and ancillary benefits of ecosystem‐based fisheries management in forage fish fisheries
Source: Ecol Appl. 2021 Aug 30;31(7):e02421. doi: 10.1002/eap.2421 (PMC9285690; doi:10.1002/eap.2421)
Supplement: Supplementary file 1 — Appendix S1 [file EAP-31-0-s001.pdf]

Supporting Information. Sanchirico, J.N., and T.E. Essington. 2021. Direct and ancillary benefits of ecosystem based fisheries management in forage fish fisheries. Ecological Applications.

Appendix S1

In the supplementary material, we present the endogenous variables in the system, parameterization and numerical methods, additional figures supporting the results in the main paper, sensitivity analysis on discount rates, forage species fish prices, pelican survival, pelican recruitment, and starting from under-exploited initial conditions for the fished species.

Contents

S1 Endogenous variables in the model **2**

S2 Parameterization and Numerical Methods **2**
S2.1 Forage Fish 2
S2.2 California Halibut 3
S2.3 Brown Pelican 4
S2.4 Economic parameters 4
S2.5 Solution Methods 7

S3 Additional Results Supporting Main Results **8**

S4 Sensitivity Analysis **15**
S4.1 Higher Discount Rate at 10% 15
S4.2 Higher Ex-vessel prices for Anchovy and Sardine 18
S4.3 Pelican Survival Independence 21
S4.4 Pelican Recruitment Independence 24
S4.5 Over-exploited Initial Conditions of the fished species 27

Notation	Description	Value	Units
$X_a(t)$	Anchovy biomass	State variable	mt km-2
$X_s(t)$	Sardine biomass	State variable	mt km-2
$X_h(t)$	Halibut biomass	State variable	mt km-2
$N_a(t)$	Anchovy Numbers	State variable	# km-2
$N_s(t)$	Sardine Numbers	State variable	# km-2
$N_h(t)$	Halibut Numbers	State variable	# km-2
$N_{bp}(t)$	Brown Pelican Numbers	State variable	# km-2
$E_a(t)$	Anchovy Fishing Effort	Control variable	Effort km-2
$E_s(t)$	Sardine Fishing Effort	Control variable	Effort km-2
$E_h(t)$	Halibut Fishing Effort	Control variable	Effort km-2

Table S1: Notation of Modeled Variables

S1 Endogenous variables in the model

Table S1 lists the endogenous variables in the system.

S2 Parameterization and Numerical Methods

This section describes the data and methods used to derive the base set of parameters of the model and describes the numerical methods.

S2.1 Forage Fish

The strategy for forage fish was to derive estimates of growth parameters, mortality, and representative biomass, and adjust recruitment so that in the absence of recruitment variation, the population biomass equals a target level reflective of time period that matches Koehn et al. (2017): (2000 – 2014). For sardine, that target biomass was 978,484 mt. Growth parameters were taken from Hill et al. (2018), specifically from the estimated relationship between mass and age (using

the intermediate curve, labelled PopS1 begin). These were then used to fit von Bertalanffy parameters:

$$w_i(t) = w_{i,\infty} + e^{-\kappa_i t}(w_{0,i} - w_{i,\infty}) \tag{S1}$$

where age at recruitment was set to the estimated $w_{0,i}$ (initial size). Natural mortality of sardine is not precisely known. The most recent stock assessment assumed M = 0.6 yr (Kuriyama et al., 2020).

For Anchovy, the target biomass, representing an average from 2000 – 2014 was 431,000mt. Growth parameters were taken from length-at-age reported by Baxter (1967), and then using length-weight conversions from fishbase (converting total length (cm) to g; a = 0.011 ; b = 2.86). The growth curve (Eq. S1) was fit to estimate $w_{0,i}$, $w_{i,\infty}$ and κ_i. Natural mortality is high for anchovies. Methot (1989) attempted to fit M using a stock assessment model but found that the data could not reliably inform M and in the end, M = 0.76 was chosen, as it allowed the model to fit the data reasonably well.

S2.2 California Halibut

We modeled only the female portion of the population. This means we are underestimating the potential top-down effect of halibut on forage fish, but this effect is already very small so likely does not drastically alter our conclusions.

Population demographics and abundance. Most estimates were derived from Maunder et al. (2011) stock assessment of California Halibut. Estimates of spawning stock biomass and recruitment were available for the Southern and Central California regions. We pooled these together to estimate average biomass (19,270 mt) and average recruitment (age 1; 3874 x 1000). We also used estimated unfished biomass (24,447). We assumed current recruitment is identical to unfished biomass, and then used equilibrium renewal method to estimate Beverton-Holt recruitment parameters (assuming steepness is 0.8, which is the median of values for Pleuronectiformes). We divided recruitment by one half to estimate female recruitment. Females are 50% mature at age 4, so we used 4 as the age of recruitment. We set M = 0.2 as per Maunder et al. (2011). Growth parameters and length-weight relationships were derived from Reed and MacCall (1988).

S2.3 Brown Pelican

Koehn et al. (2017) derives an estimate of total brown pelicans abundance in the entire California current (26,000 individuals). Because here we assume that pelican do not affect the other model state variables, we instead model them as a proportion of initial density (so we set initial density to 1). Determining biological thresholds is unclear, as any federal ESA listing would look at the entire range of the California Sub Species which resides from the California Current into the Gulf of Mexico and further south to central Mexico. IUCN listing of vulnerable would normally be made if the population exhibits a greater than 50% decline, if the causes are known and have been ceased, or a decline greater than 30% otherwise (IUCN, 2001). Currently, the entire subspecies abundance is well above the original criteria for delisting (3,000 breeding pairs, while Anderson et al. (2013) calculates over 70,000 breeding pairs in 2006).

S2.4 Economic parameters

We calibrate the economic parameters (prices, catchability, and costs) to the Pacific Sardine, Northern Anchovy, and California Halibut fisheries. Ex-vessel prices for each of the species are in year 2015 dollars. Anchovy data are from 1980-1997, where we utilize the maximum ex-vessel price of $\$0.109$ per lb (PFMC (1998)). For sardines, we use $\$0.094$ per lb, which is the maximum sardine ex-vessel value from 1990-2011 (CDFW (2013b)). The range of California Halibut ex-vessel prices over the 1990-2011 period are $\$1.09 - \$4.69$ per lb (CDFW (2013a)). We use $\$3.39$ per lb. in our base case. Second, to determine the cost and catchability parameters (3 per species), we utilized the following conditions: (1) average profits in each fishery were set equal to zero (long-run open access condition); (2) matched the range of plausible fishing mortality rates in the fisheries at our initial conditions on the fished stocks; and (3) fishing remains profitable when the biomass of sardines, anchovy, and Halibut are at or above 30% of unfished biomass levels in the no variability case (unfished biomass determined by model parameters). This latter condition is important in the cases of growth variability as the natural swings in populations could bring the populations below this level. The condition is also consistent with the calls to maintain forage stocks at 40% of unfished biomass, which would not be an issue if fishing was unprofitable at levels of the population below that threshold. We also assume a discount rate of 4% at the base case. See Table S3 for specific levels.

Notation	Description	Value	Units	Source
r_i	avg. recruitment of sardine or anchovy	Varies	number	assumed / scenario
A	amplitude of log recruitment deviations	2	unitless	assumed / scenario
ω_{ri}	weight of recruit for anchovy, sardine, and halibut, respectively	.01E-3,.01E-3,0.0013	mt	
κ_i	catabolic rate term for anchovy, sardine, and halibut, respectively	0.13, 0.215, .12	yr-1	Baxter (1967); Kuriyama et al. (2020)
M_a	natural mortality rate of anchovy	0.76	yr-1	Methot (1989)
M_s	natural mortality rate of sardine	0.6	yr-1	Kuriyama et al. (2020)
M_p	natural mortality rate of adult halibut	0.2	yr-1	Maunder et al. (2011)
M_{pj}	natural mortality rate of juv. halibut	0.2	yr-1	Maunder et al. (2011)
M_{bp}	natural mortality rate of anchovy	0.22	yr-1	Punt et al. (2016)
C_{max}	max. mass specific consumption rate	9.27	g/g/yr-1	Holsman et al. (2019)
α_a	eff. rate of search and capture on A		yr-1	derived
α_s	eff. rate of search and capture on S		yr-1	derived
Y	per capita feeding on other prey		mt yr-1	derived
P_i	predation mortality rate on prey i		yr-1	derived
p_i	period of recruitment fluctuation for prey species i	*	yr	assumed / scenario
s_i	starting point for sin wave for species i		degrees	
d	allometric scaling of consumption	0.75	unitless	Essington et al. (2001)
a	maximum recruitment rate (Age 1 Fm.)	1.25E+03	mt-1	Maunder et al. (2011)
b	density dependent Beverton Holt eq.	6.06E-04	mt-1	Maunder et al. (2011)
θ	assimilation efficiency of halibut	0.65	unitless	Holsman et al. (2019)
θ_{1r}	value of prey depletion that makes f =0	0.2	unitless	Punt et al. (2016)
θ_{2r}	reference level of prey depletion	0.4	unitless	Punt et al. (2016)
θ_{3r}	value of f when prey depletion = θ_{2r}	0.95	unitless	Punt et al. (2016)
θ_{1s}	value of prey depletion that makes f =0	0	unitless	Punt et al. (2016)
θ_{2s}	reference level of prey depletion	0.2	unitless	Punt et al. (2016)
θ_{3s}	value of f when prey depletion = θ_{2s}	0.95	unitless	Punt et al. (2016)

Table S2: Ecological Parameters

Notation	Description	Value	Units	Source
$\tilde{p}_a$	Ex-vessel price for Anchovy	254.20	$ per mt	PFMC (1998)
$\tilde{p}_s$	Ex-vessel price for Sardine	206.3	$ per mt	CDFW (2013b)
$\tilde{p}_h$	Ex-vessel price for Halibut	7480.00	$ per mt	CDFW (2013a)
c_{a1}	Linear cost of effort for Anchovy	25.3256	$ per effort	calibrated
c_{s1}	Linear cost of effort for Sardine	33.0334	$ per effort	calibrated
c_{h1}	Linear cost of effort for Halibut	36.7500	$ per effort	calibrated
c_{a2}	Quadratic cost of effort for Anchovy	4.485	$ per effort^2	calibrated
c_{s2}	Quadratic cost of effort for Sardine	4.485	$ per effort^2	calibrated
c_{h2}	Quadratic cost of effort for Halibut	6.500	$ per effort^2	calibrated
q_a	Catchability coefficient for Anchovy	.2588	1/effort*time	calibrated
q_s	Catchability coefficient for Sardine	.2012	1/effort*time	calibrated
q_h	Catchability coefficient for Halibut	.1725	1/effort*time	calibrated

Table S3: Economic Parameters

Notation	Description	Units	Level
$X_a(0)$	Anchovy biomass at time 0	mt km-2	3.3113
$X_s(0)$	Sardine biomass at time 0	mt km-2	16.5563
$X_h(0)$	Halibut biomass at time 0	mt km-2	10.1235
$N_a(0)$	Anchovy Numbers at time 0	# km-2	1.3245
$N_s(0)$	Sardine Numbers at time 0	# km-2	2.4834
$N_h(0)$	Halibut Numbers at time 0	# km-2	.0586
$N_{bp}(0)$	Brown Pelican Numbers at time 0	# km-2	100
T	Time Horizon	Years	40
N	Collocation nodes		110
ψ	Parameter Eq. 16		0 to .25

Table S4: Optimization Parameters

S2.5 Solution Methods

We solve each model variant using a direct optimization approach based on pseudo-spectral collocation (Benson et al., 2006). This involves approximating the state and control variables using a collection of basis functions over a finite time ($t \in [0, T]$). The method is referred to as direct because the approximated states and controls are used to form a constrained nonlinear program (NLP). In this problem, the objective (e.g., the integral in Eq. 15) is maximized, subject to constraints including initial conditions, the state equation, and when appropriate the constraint on pelican populations. Examples of a bioeconomic application of pseudo-spectral collocation with an extended discussion of the numerical details is provided by Sanchirico and Springborn (2011), Kling et al. (2016), and Essington et al. (2018).

Pseudospectral collocation was implemented using TOMLAB (v. 7.8) (Holmström, 1999) and the accompanying PROPT software module (Rutquist and Edvall, 2010). The approximate NLP is solved using the general-purpose nonlinear optimization package SNOPT (Gill et al., 2006). The algorithm ensures that the residual error of the constraints is minimized at the collocation points. An advantage of this approach over indirect methods (e.g., shooting) is that we can directly incorporate the non-negativity constraints into the problem (Judd, 1998). This feature enables us to find optimal solutions that might reside on the boundary of the

control set for a period of time, which as we find is a critical piece of our optimal solutions especially in the presence of environmental variability.

Another advantage is the ability to incorporate a time delay in a straightforward manner, where we assume that the initial conditions hold for the periods before $t = 0$ (e.g., if the age at maturation is equal, then we have $t(-4), t(-3), t(-2), t(-1)$ set to the initial conditions). We relaxed this assumption by burning in the model results (going backwards in time with assumed fishing mortality rate and our initial conditions using a delay-difference 4th order Runga-Kutta algorithm). The results were qualitatively similar but not as robust as with the assumption of constant levels.

Because of the non-linearity, large state space, and time delay in the population dynamic system, we employed a number of steps to ensure that algorithm solutions were optimal. First, we generated good initial guesses by solving the model without the time delay. These solutions were then used as the initial guess with the time delay. Second, we solved the model from a number of different initial conditions. If we are finding the optimal solution, then these solutions should all reach the same long-run path after an initial adjustment period. Finally, we also solved the model using different numbers of collocation points and terminal times to ensure that the solutions are robust to different degrees of polynomials and time intervals. We report out results with 110 collocation nodes and $T = 40$ (all equilibrium dynamic paths were reached in the first 20 periods).

S3 Additional Results Supporting Main Results

Table S5: Impacts of Pelican Constraint

Regime	No Variability			Synchronous			Asynchronous		
	T_{cross}	T_{change}	$\epsilon_{NPV/P}$	T_{cross}	T_{change}	$\epsilon_{NPV/P}$	T_{cross}	T_{change}	$\epsilon_{NPV/P}$
A	30.53	4.39	1.42	32.83	5.51	0.61	31.01	5.12	0.83
S	30.04	5.12	2.48	32.83	5.51	1.40	30.53	8.52	2.11
H	0.00	0.00		0.00	0.00		0.00	0.00	
A+H	31.01	4.04	0.86	33.26	5.51	0.33	31.01	4.75	0.59
A+S	30.53	5.51	1.97	32.83	5.51	0.54	31.01	5.91	1.04
S+H	29.55	5.91	2.40	32.83	5.91	1.02	30.53	8.52	2.25
A+S+H	31.01	4.39	0.99	33.26	6.32	0.15	31.01	5.12	0.80

These results correspond to the base case parameters with $\Psi = .2$. T_{cross} is the time at which the optimal solution without the 20% increase in $N_{bp}(t)$ imposed would cross the threshold. T_{change} is the time at which the pelican population level in the optimal solution with the constraint deviates from the optimal solution without the constraint imposed. $\epsilon_{NPV/P}$ is the elasticity of net present value with an increase in pelican years, which provides a unitless measure of the responsiveness in the cost of meeting the constraint (reduction in net present value) with an increase in the total pelican years. An elasticity greater than one implies that a 1% change in the pelican numbers results in a larger than 1% decrease in net present value.

Table S6: Catch control Rules and Optimal Management

Variability	Harvest Control Rule		Hockey Stick	
	ΔNPV_S	ΔNPV_{S+A+H}	ΔNPV_S	ΔNPV_{S+A+H}
No Variability	81.05	89.62	97.39	78.09
Synchronous	82.94	90.94	64.00	80.88
Anti-synchronous	79.02	89.55	55.72	77.94

These results correspond to the base case parameters with $\Psi = .2$. ΔNPV_i represents the percent reduction in net present value (cost of imposing the catch control rule) of the catch control rule relative to the management regime, i.

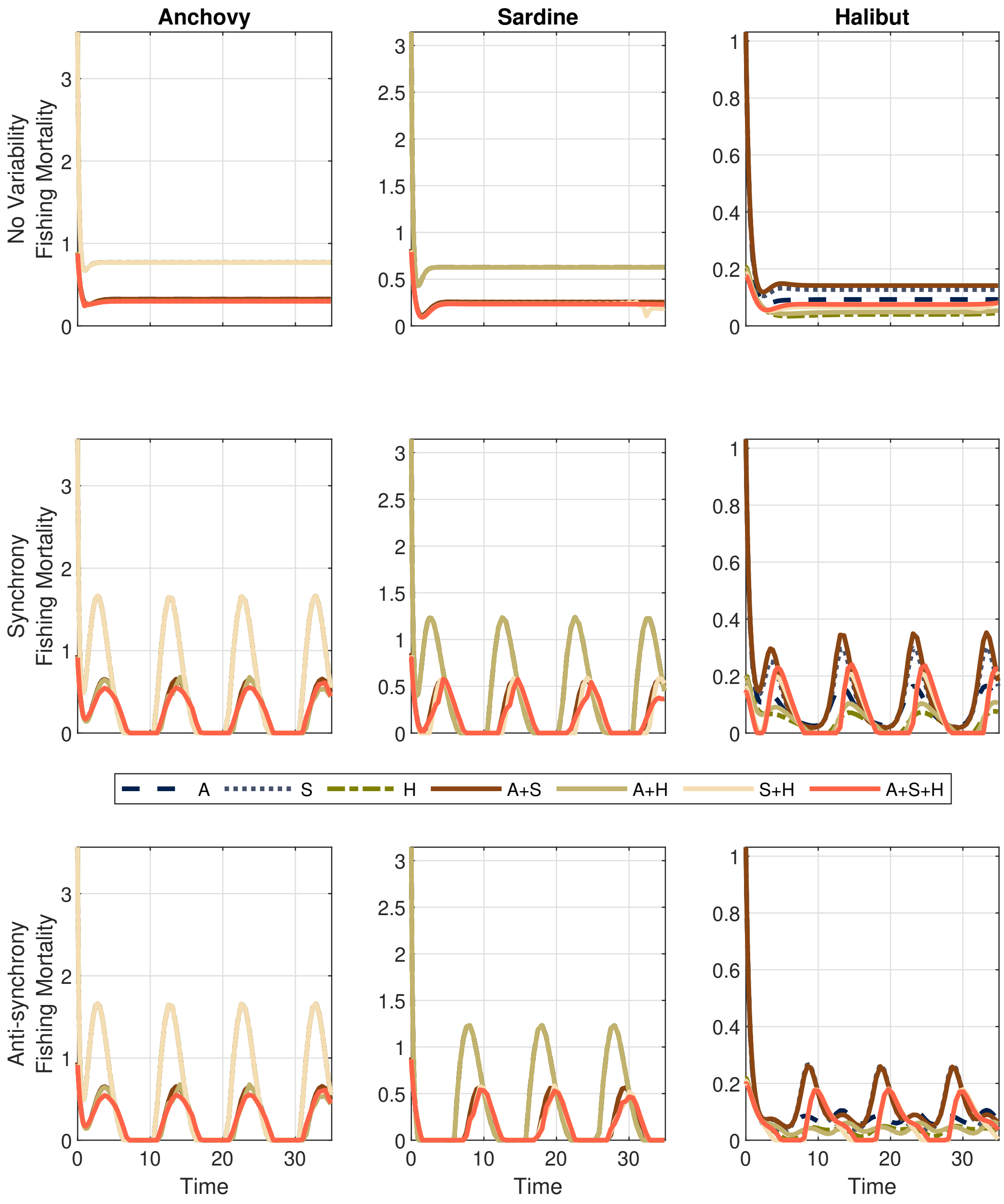

Figure S1: Fishing Mortality Dynamics for the management regime-variability pairs. Management regime labels are found in Table 1. The top row corresponds to no variability, middle row to synchronous variability and the bottom row to anti-synchronous. The first column is Anchovy, second column is Sardine, and third column is Halibut.

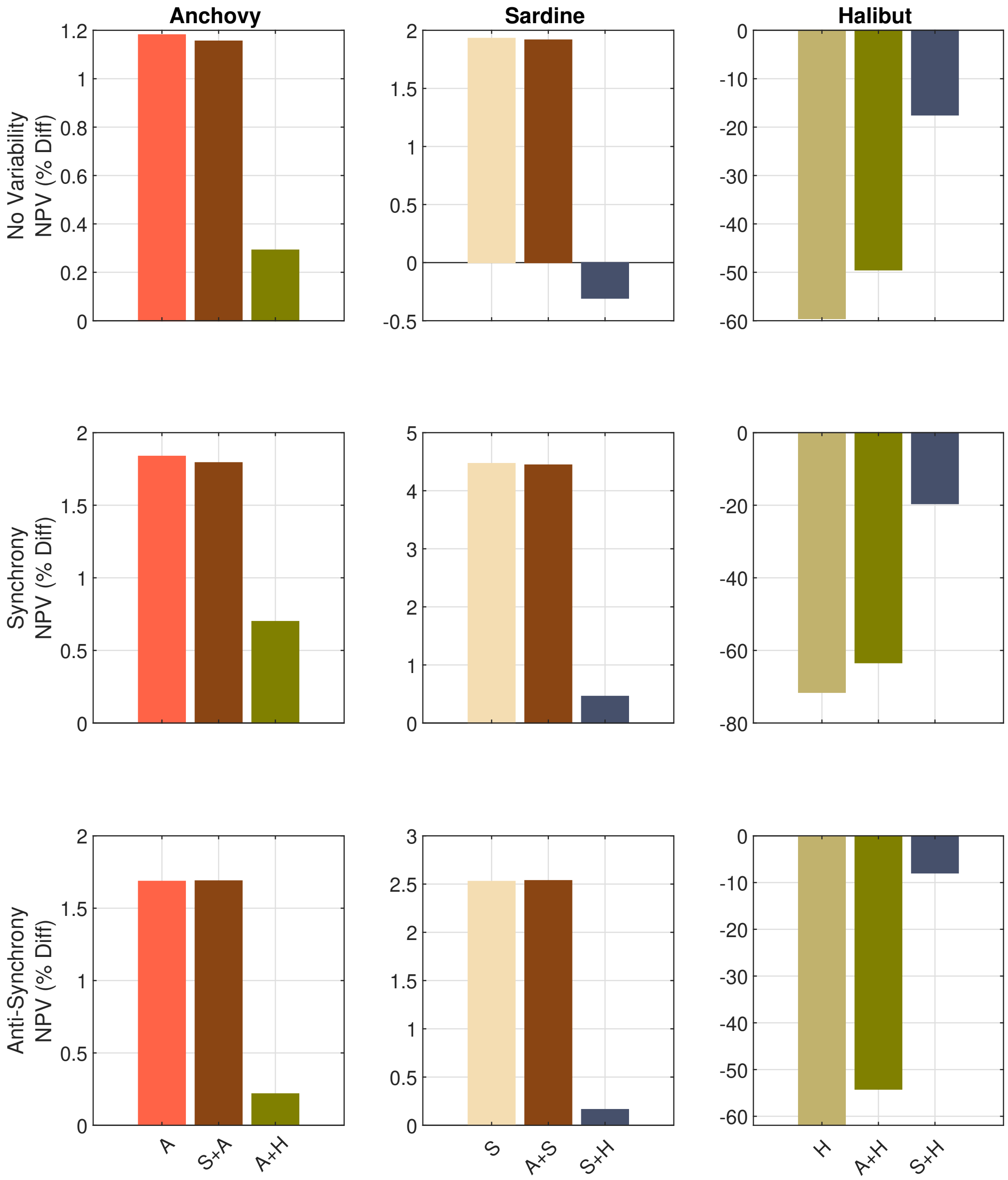

Figure S2: Net present value for each species under different management regimes relative to the full-EBFM approach. Management regime labels are found in Table 1. The top row corresponds to no variability, middle row to synchronous variability and the bottom row to anti-synchronous. The first column is Anchovy, second column is Sardine, and third column is Halibut. A positive percent difference corresponds to the species has higher net present value when it is not managed as part of the full-EBFM case.

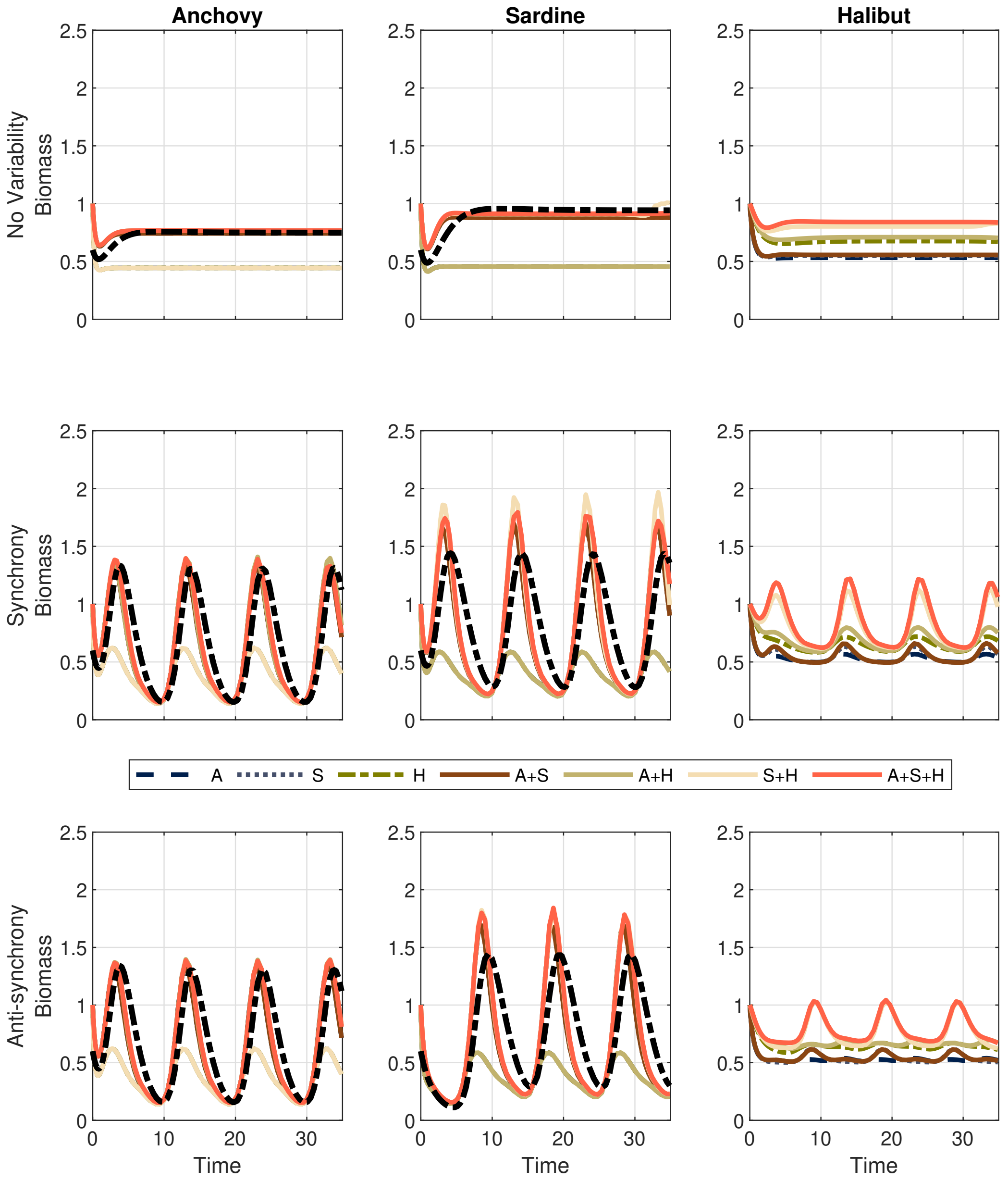

Figure S3: Dynamics of Biomass with 40% of unfished biomass. Biomass is scaled off of the initial conditions to facilitate comparisons across the panels. Management regime labels are found in Table 1. The top row corresponds to no variability, middle row to synchronous variability and the bottom row to anti-synchronous. The first column is Anchovy, second column is Sardine, and third column is Halibut. The black dashed line is 40% of unfished biomass levels at each point in time. We only represent the line for the forage fish species.

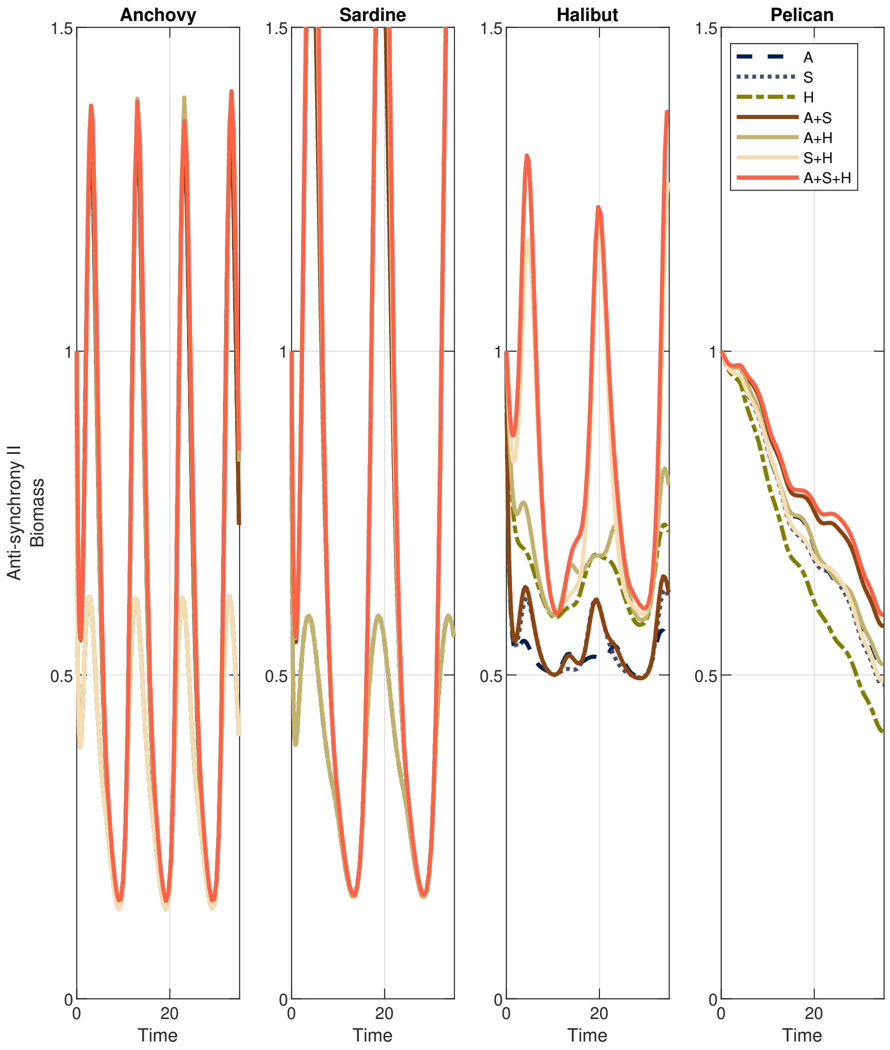

Figure S4: Optimal biomass levels over time across the management regimes for anti-synchronous due to Sardine having different frequency. Biomass is scaled off of the initial conditions to facilitate comparisons across the panels. Management regime labels are found in Table 1. The first column is Anchovy, second column is Sardine, third column is Halibut, and fourth is Pelican.

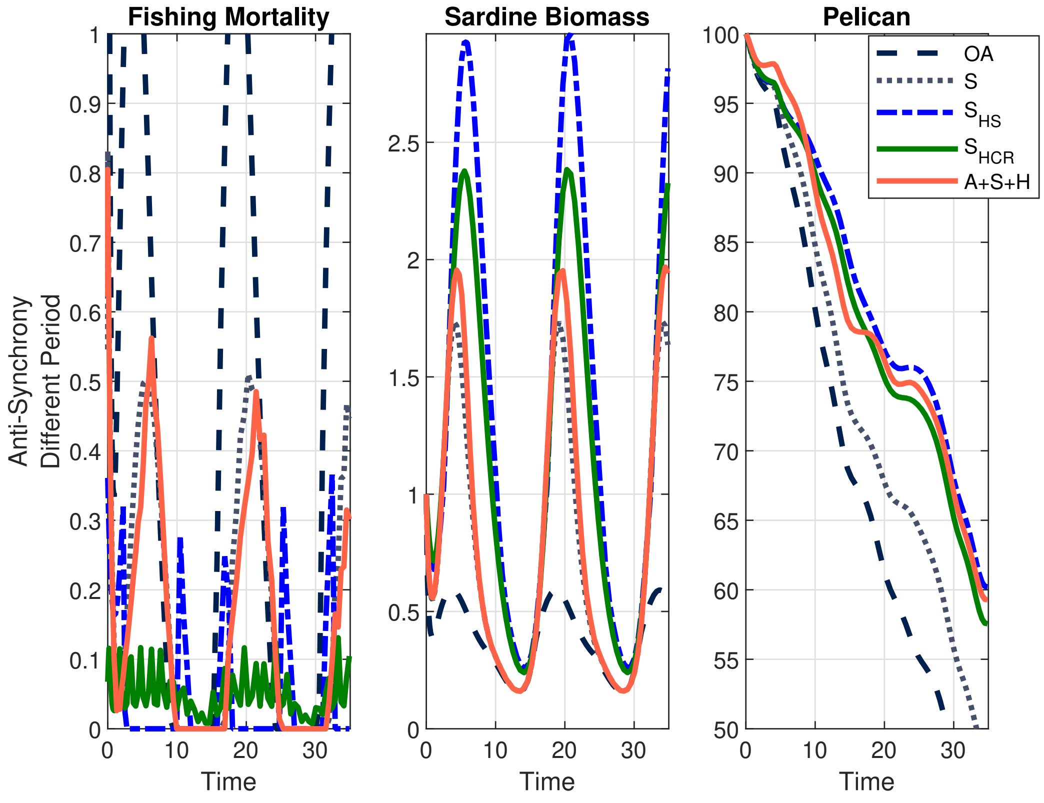

Figure S5: Sardine Management and Pelican Numbers. OA corresponds to open-access, S corresponds to optimal management of sardine only, S_{HS} is the hockey-stick formulation for the catch control rule, S_{HCR} is the harvest control rule, and A+S+H is the full-EBFM optimal. The first column are fishing mortality rates, second column is biomass levels, and the third column are pelican numbers over time.

S4 Sensitivity Analysis

S4.1 Higher Discount Rate at 10%

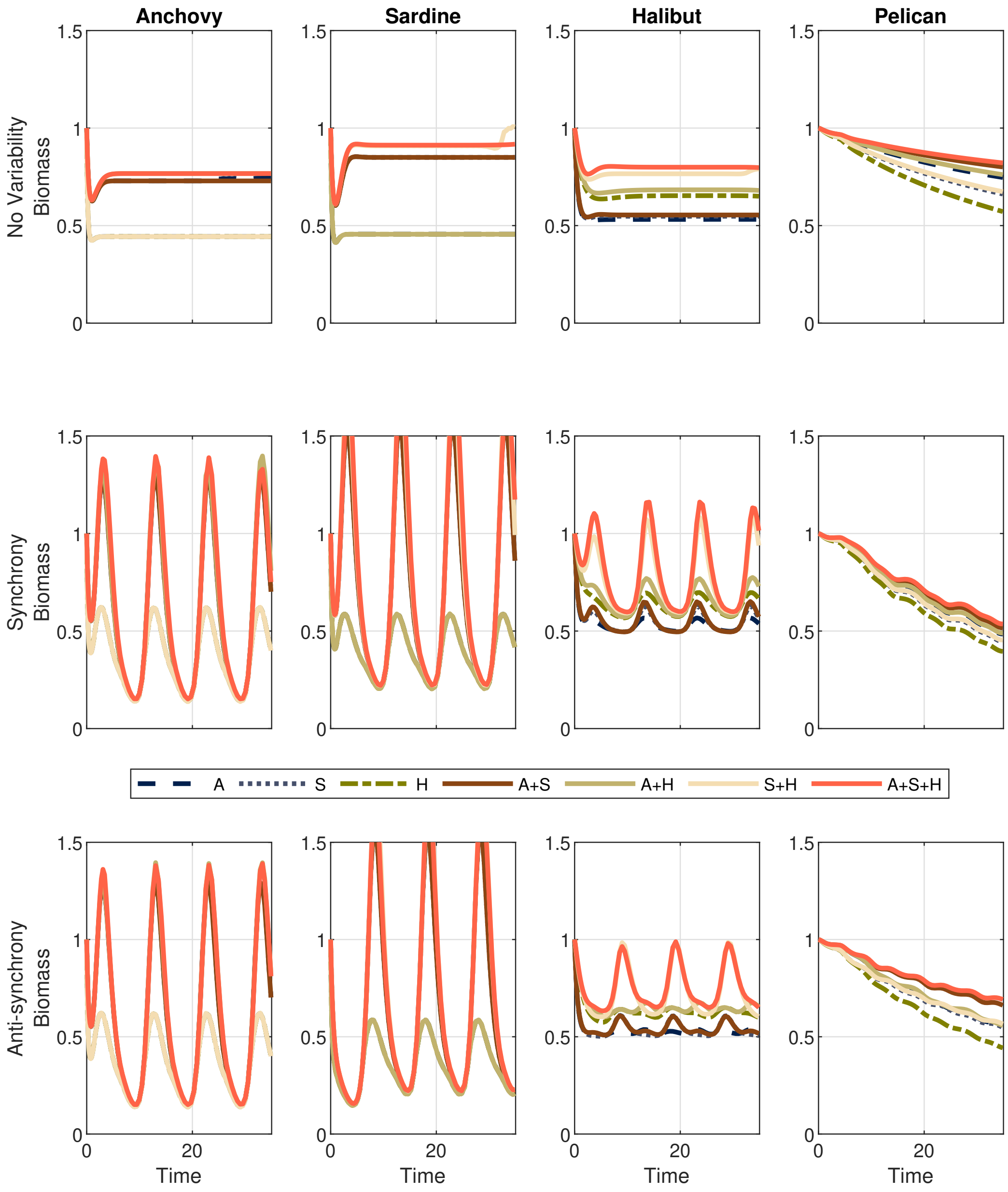

Figure S6: Biomass Dynamics for the management regime-variability pairs. Biomass is scaled off of the initial conditions to facilitate comparisons across the panels. Management regime labels are found in Table 1. The top row corresponds to no variability, middle row to synchronous variability and the bottom row to anti-synchronous. The first column is Anchovy, second column is Sardine, third column is Halibut, and fourth column is Pelican.

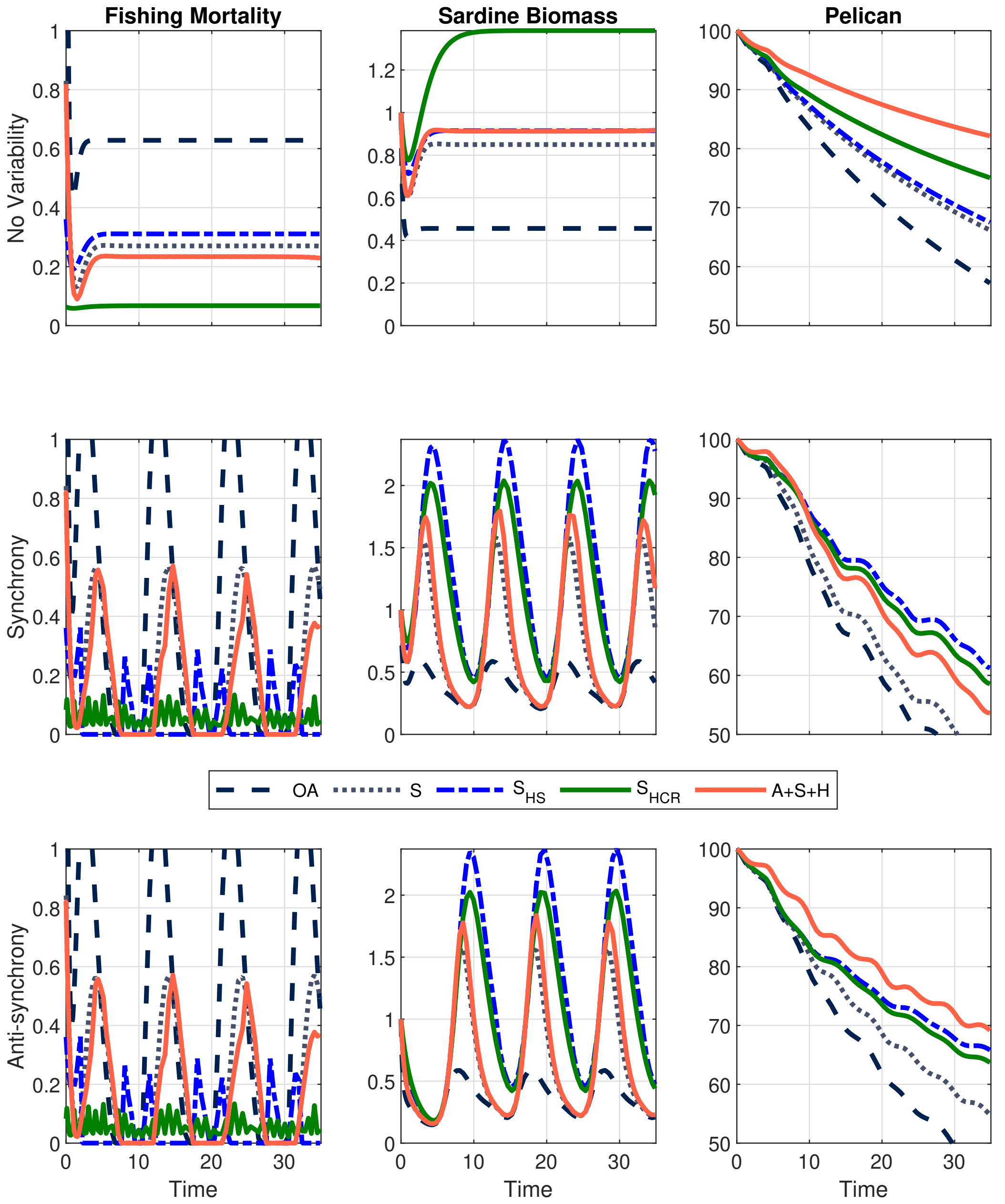

Figure S7: Sardine Management and Pelican Numbers. The legend is as follows: OA corresponds to open-access, S corresponds to optimal management of sardine only, S_{HS} is the hockey-stick formulation for the catch control rule, S_{HCR} is the harvest control rule, and A+S+H is the full-EBFM optimal. The first column are fishing mortality rates, second column is biomass levels, and the third column are pelican numbers over time. The top row is the no variability case, middle row is synchronous and bottom row is anti-synchronous.

S4.2 Higher Ex-vessel prices for Anchovy and Sardine

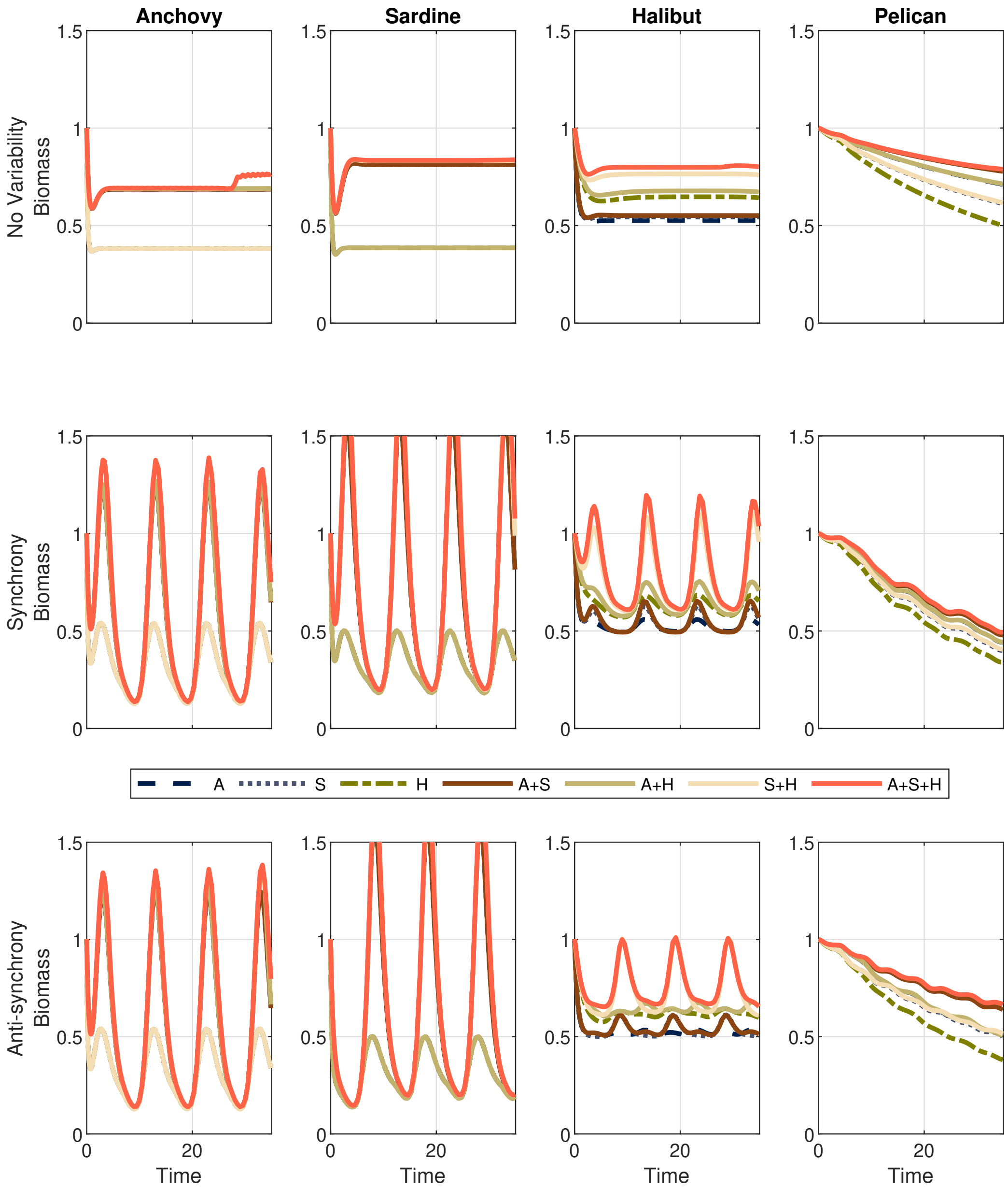

Figure S8: Biomass Dynamics for the management regime-variability pairs. Biomass is scaled off of the initial conditions to facilitate comparisons across the panels. Management regime labels are found in Table 1. The top row corresponds to no variability, middle row to synchronous variability and the bottom row to anti-synchronous. The first column is Anchovy, second column is Sardine, third column is Halibut, and fourth column is Pelican.

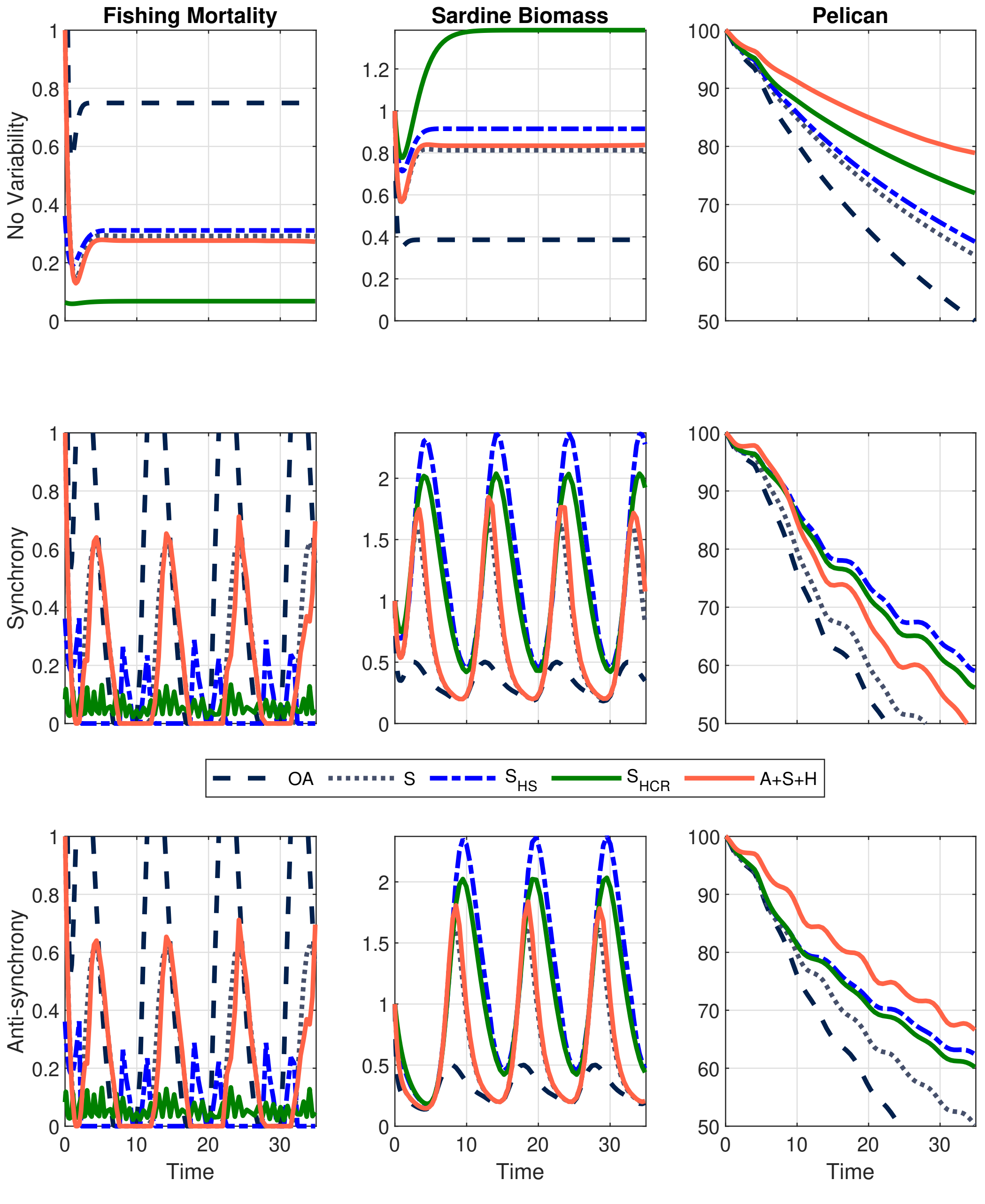

Figure S9: Sardine Management and Pelican Numbers. The legend is as follows: OA corresponds to open-access, S corresponds to optimal management of sardine only, S_{HS} is the hockey-stick formulation for the catch control rule, S_{HCR} is the harvest control rule, and A+S+H is the full-EBFM optimal. The first column are fishing mortality rates, second column is biomass levels, and the third column are pelican numbers over time. The top row is the no variability case, middle row is synchronous and bottom row is anti-synchronous.

S4.3 Pelican Survival Independence

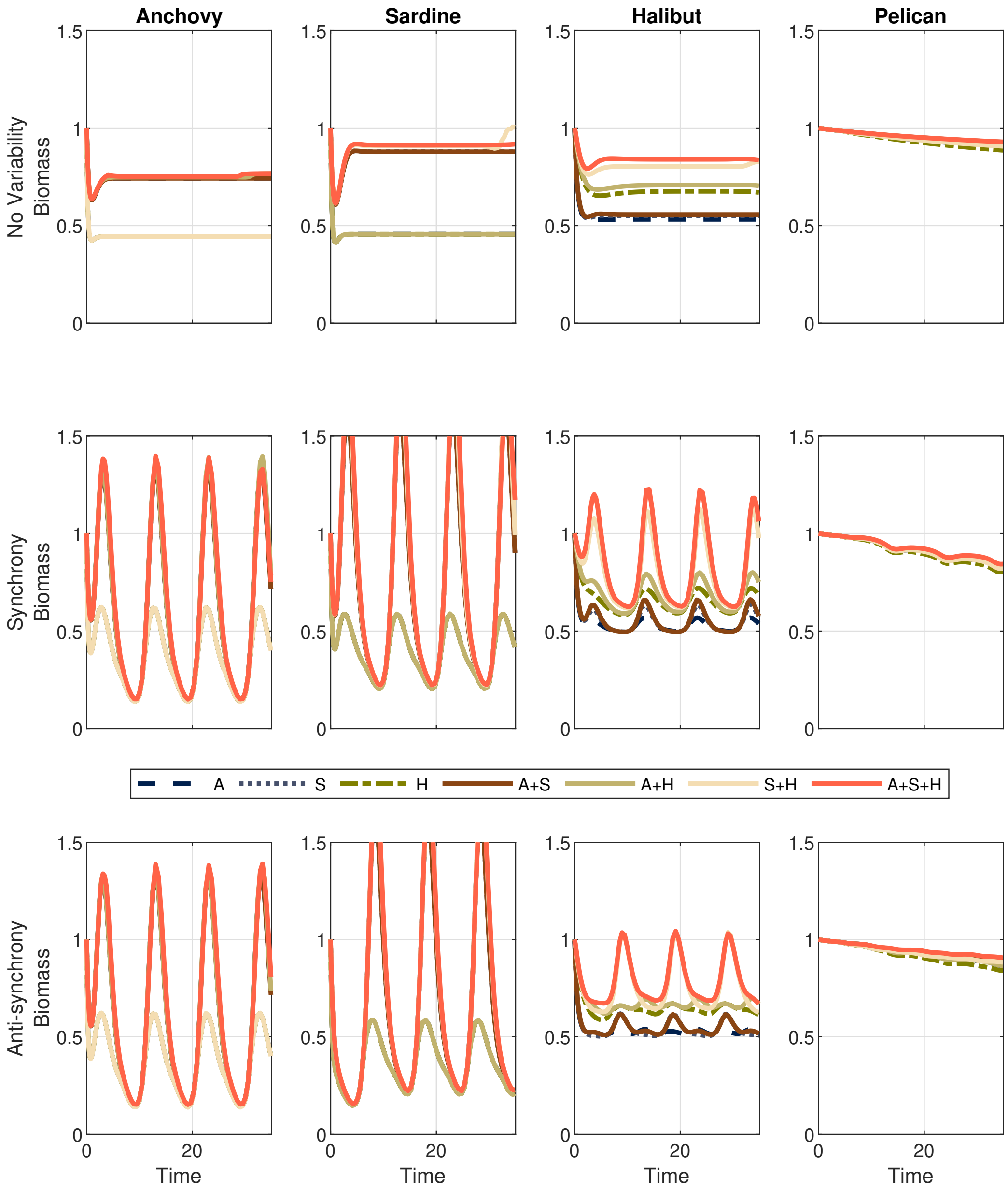

Figure S10: Biomass Dynamics for the management regime-variability pairs. Biomass is scaled off of the initial conditions to facilitate comparisons across the panels. Management regime labels are found in Table 1. The top row corresponds to no variability, middle row to synchronous variability and the bottom row to anti-synchronous. The first column is Anchovy, second column is Sardine, third column is Halibut, and fourth column is Pelican.

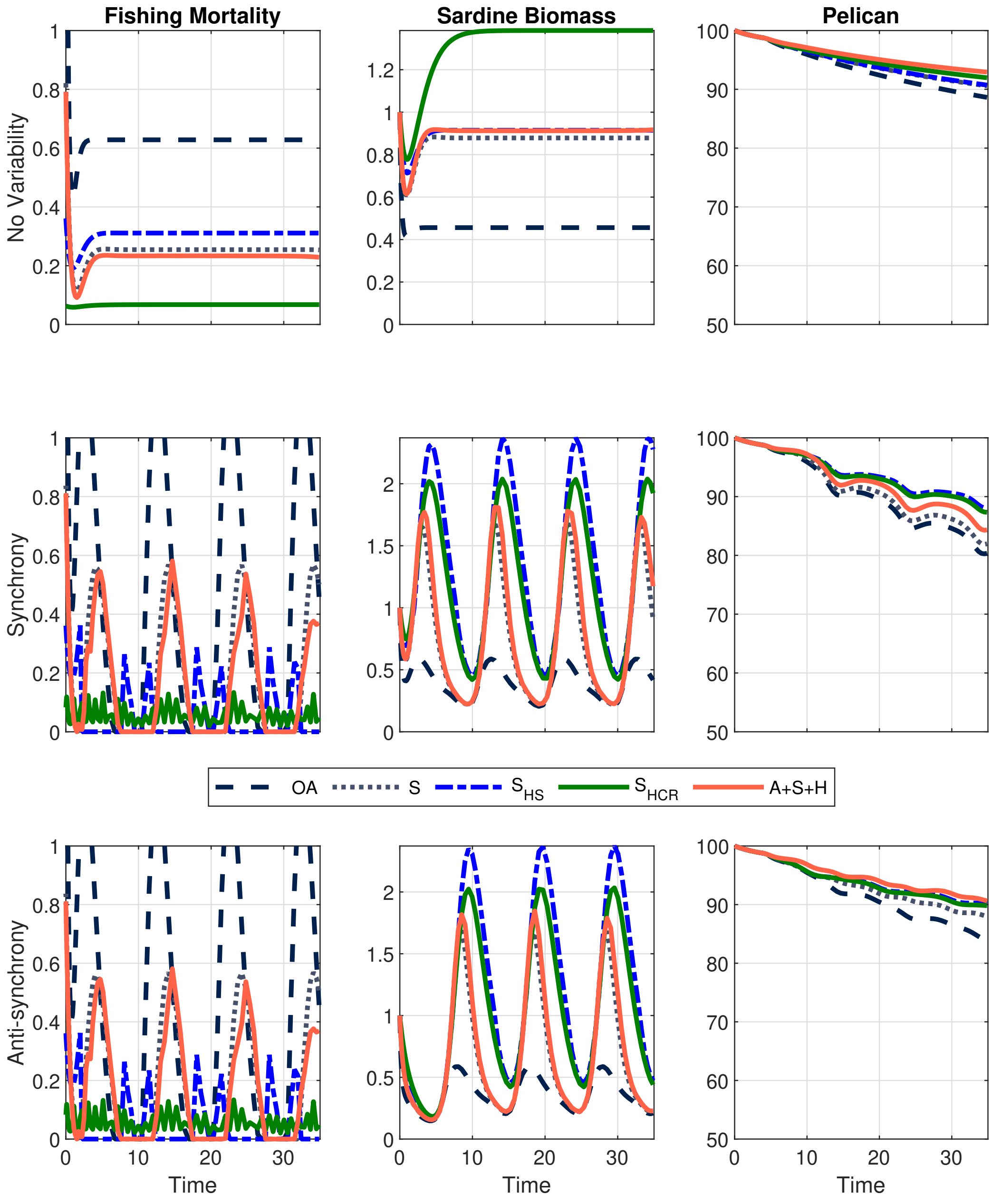

Figure S11: Sardine Management and Pelican Numbers. The legend is as follows: OA corresponds to open-access, S corresponds to optimal management of sardine only, S_{HS} is the hockey-stick formulation for the catch control rule, S_{HCR} is the harvest control rule, and A+S+H is the full-EBFM optimal. The first column are fishing mortality rates, second column is biomass levels, and the third column are pelican numbers over time. The top row is the no variability case, middle row is synchronous and bottom row is anti-synchronous.

S4.4 Pelican Recruitment Independence

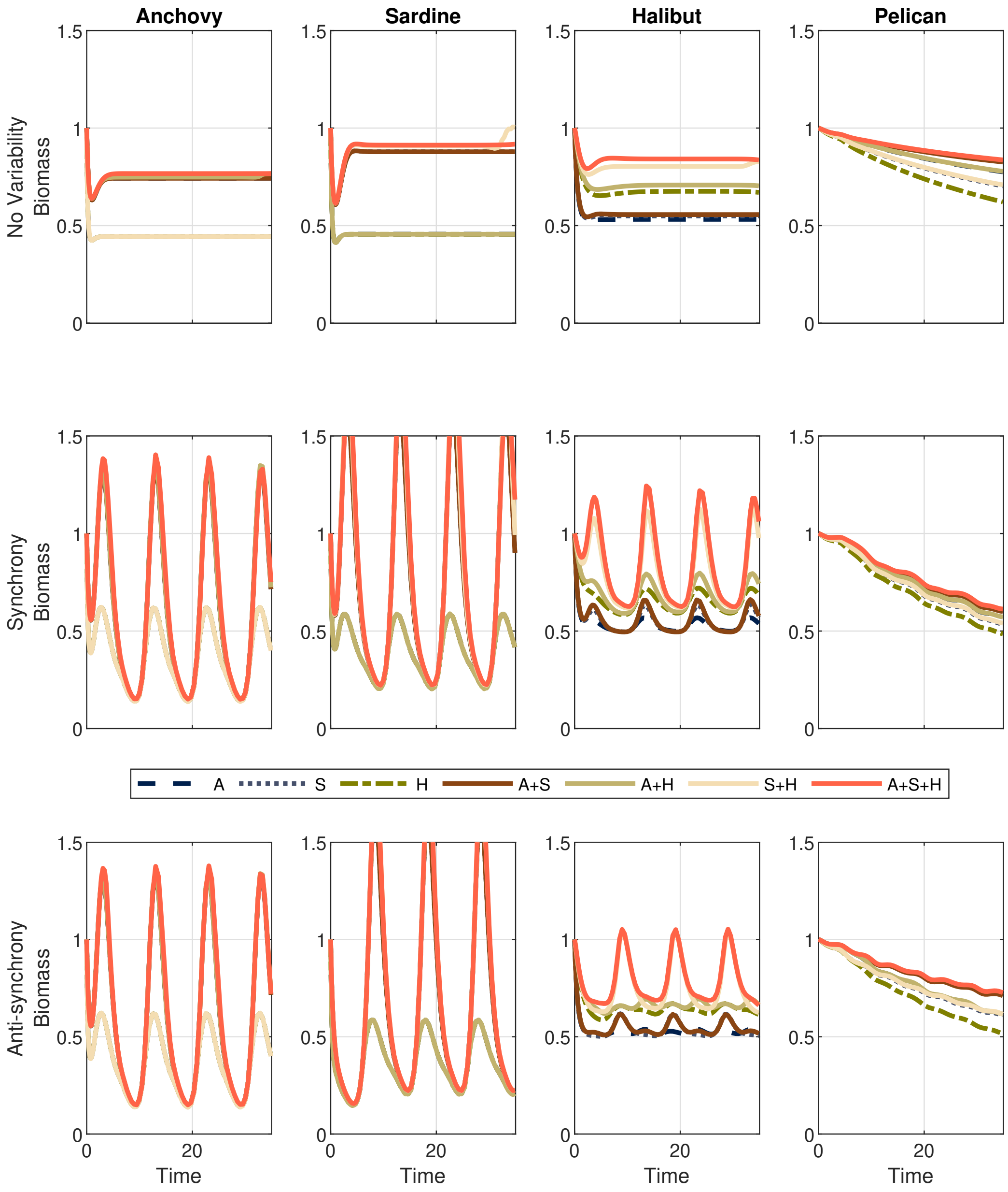

Figure S12: Biomass Dynamics for the management regime-variability pairs. Biomass is scaled off of the initial conditions to facilitate comparisons across the panels. Management regime labels are found in Table 1. The top row corresponds to no variability, middle row to synchronous variability and the bottom row to anti-synchronous. The first column is Anchovy, second column is Sardine, third column is Halibut, and fourth column is Pelican.

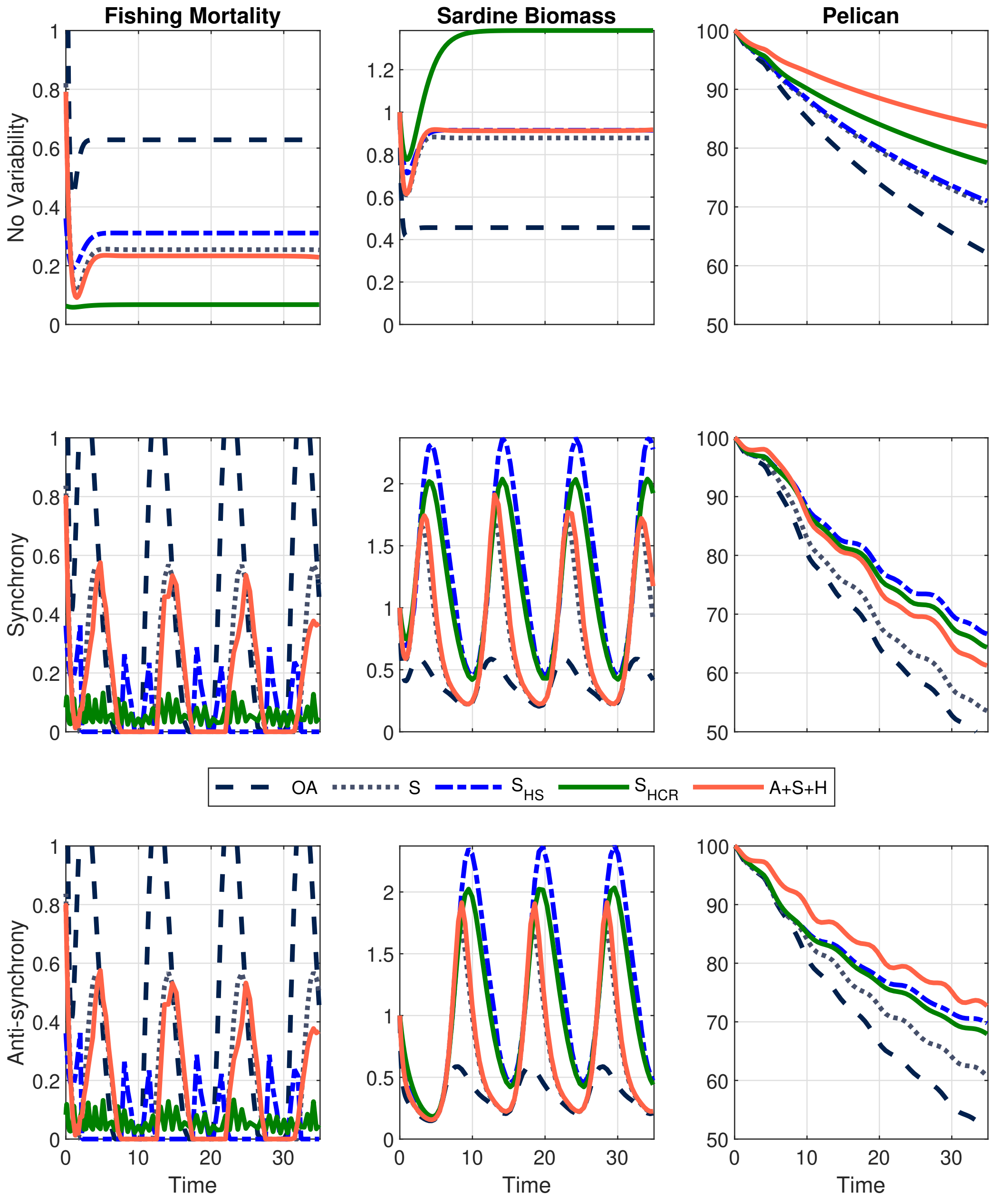

Figure S13: Sardine Management and Pelican Numbers. The legend is as follows: OA corresponds to open-access, S corresponds to optimal management of sardine only, S_{HS} is the hockey-stick formulation for the catch control rule, S_{HCR} is the harvest control rule, and A+S+H is the full-EBFM optimal. The first column are fishing mortality rates, second column is biomass levels, and the third column are pelican numbers over time. The top row is the no variability case, middle row is synchronous and bottom row is anti-synchronous.

S4.5 Over-exploited Initial Conditions of the fished species

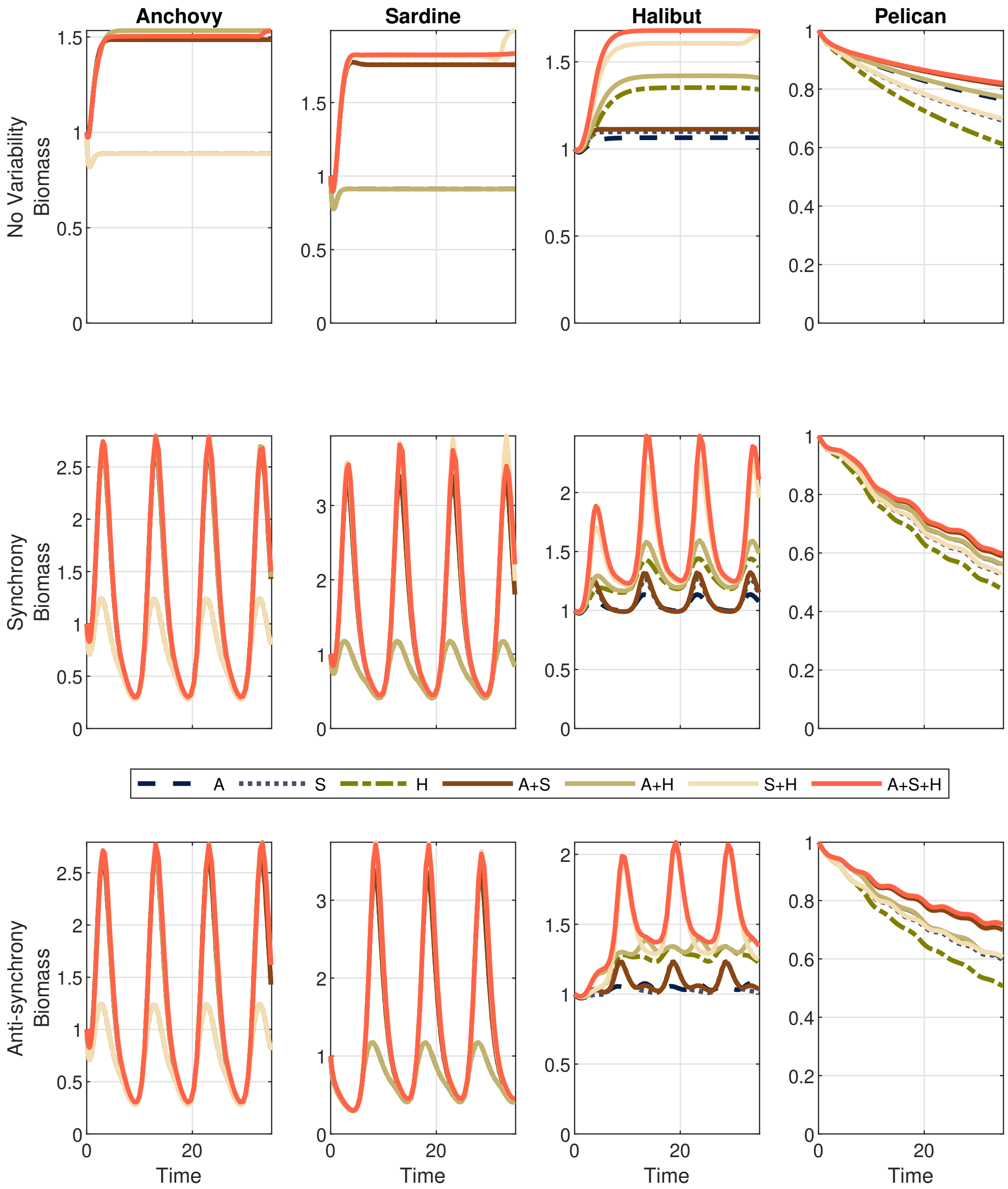

Figure S14: Biomass Dynamics for the management regime-variability pairs. Biomass is scaled off of the initial conditions to facilitate comparisons across the panels. Management regime labels are found in Table 1. The top row corresponds to no variability, middle row to synchronous variability and the bottom row to anti-synchronous. The first column is Anchovy, second column is Sardine, third column is Halibut, and fourth column is Pelican.

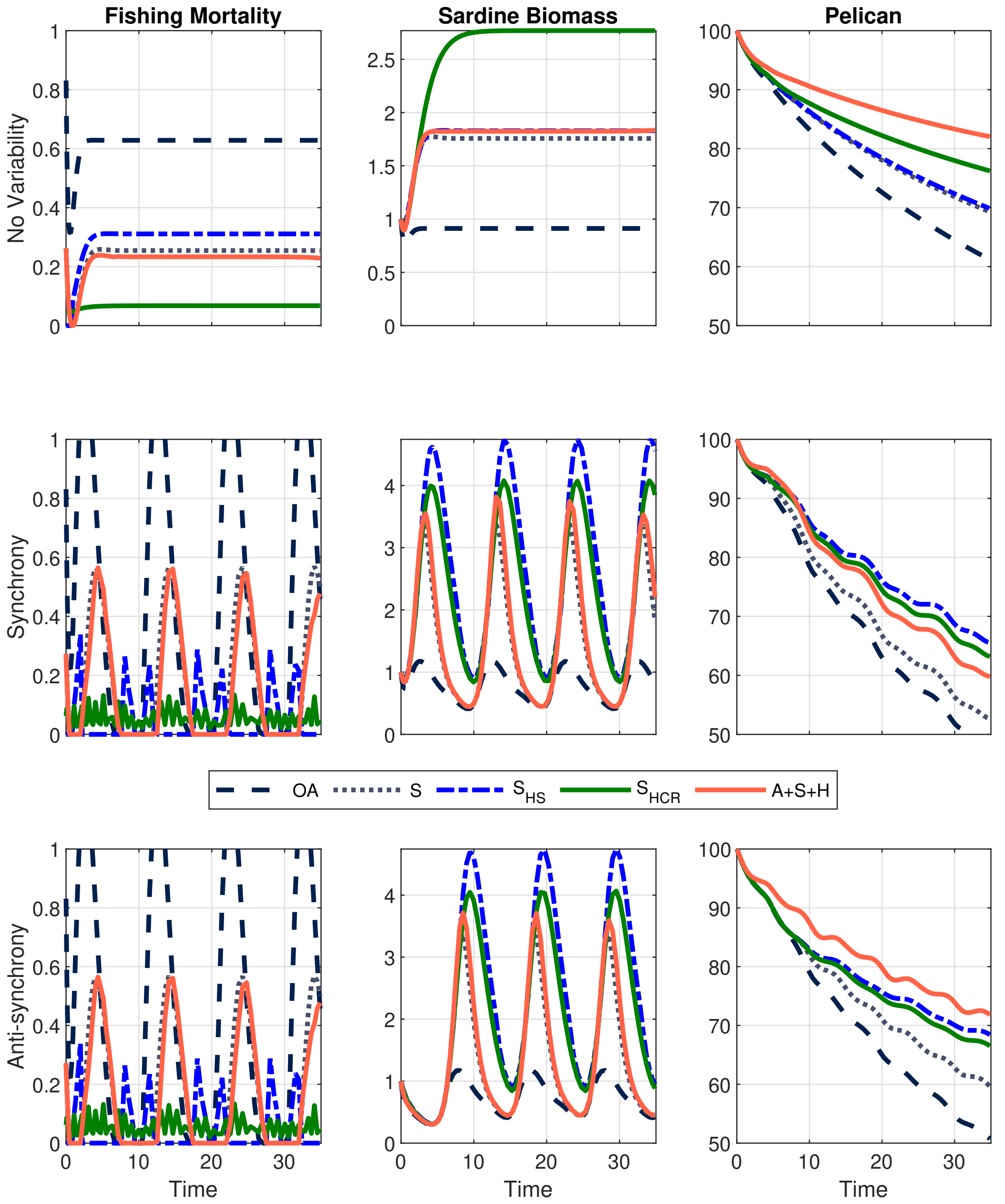

Figure S15: Sardine Management and Pelican Numbers. The legend is as follows: OA corresponds to open-access, S corresponds to optimal management of sardine only, S_{HS} is the hockey-stick formulation for the catch control rule, S_{HCR} is the harvest control rule, and A+S+H is the full-EBFM optimal. The first column are fishing mortality rates, second column is biomass levels, and the third column are pelican numbers over time. The top row is the no variability case, middle row is synchronous and bottom row is anti-synchronous.

References

D. W. Anderson, C. J. Henny, C. GODíNEZ-REYES, F. Gress, E. L. Palacios, K. S. D. Prado, J. P. Gallo-Reynoso, and J. Bredy. Size and distribution of the California Brown Pelican metapopulation in a non-ENSO year. *Marine Ornithology*, 41:95–106, Oct. 2013. ISSN 1018-3337, 2074-1235. URL `http://www.marineornithology.org/content/get.cgi?ident=41_2_95-106`.

J. L. Baxter. Summary of biological information on the Northern anchovy Engraulis mordax Girard. *California Cooperative Oceanic Fisheries Investigations Reports*, 11:110–116, 1967.

D. A. Benson, G. T. Huntington, T. P. Thorvaldsen, and A. V. Rao. Direct trajectory optimization and costate estimation via an orthogonal collocation method. *Journal of Guidance, Control, and Dynamics*, 29(6):1435–1440, 2006.

CDFW. Status of the fisheries report, an update through 2011: California halibut. *California Department of Fish and Wildlife*, 2013a.

CDFW. Status of the fisheries report, an update through 2011: Pacific sardine. *California Department of Fish and Wildlife*, 2013b.

T. Essington, J. Sanchirico, and M. Baskett. Economic value of ecological information in ecosystem-based natural resource management depends on exploitation history. *Proceedings of the National Academy of Sciences of the United States of America*, 115(7), 2018. ISSN 10916490. doi: 10.1073/pnas.1716858115.

T. E. Essington, J. F. Kitchell, and C. J. Walters. The von bertalanffy growth function, bioenergetics, and the consumption rates of fish. *Canadian Journal of Fisheries and Aquatic Sciences*, 58(11):2129–2138, 2001. ISSN 12057533 0706652X. doi: 10.1139/cjfas-58-11-2129.

P. E. Gill, W. Murray, and M. A. Saunders. User's guide for snopt version 7: Software for large-scale nonlinear programming, systems optimization laboratory (sol), 2006.

K. T. Hill, P. R. Crone, and J. P. Zwolinsky. Assessment of the pacific sardine resource in 2018 for u.s. management in 2018 - 2019. Technical report, NOAA-Fisheries, 2018.

K. Holmström. The tomlab optimization environment in matlab. 1999.

K. K. Holsman, K. Aydin, J. Sullivan, T. Hurst, and G. H. Kruse. Climate effects and bottom-up controls on growth and size-at-age of Pacific halibut (Hippoglossus stenolepis) in Alaska (USA). *Fisheries Oceanography*, 28(3): 345–358, 2019. ISSN 1365-2419. doi: 10.1111/fog.12416. URL https://onlinelibrary.wiley.com/doi/abs/10.1111/fog.12416.

IUCN. Iucn red list categories and criteria: Version3.1. *http://www. iucnredlist. org/documents/redlist_cats_crit_en. pdf*, 2001.

K. L. Judd. *Numerical methods in economics*. MIT press, 1998.

D. M. Kling, J. N. Sanchirico, and J. E. Wilen. Bioeconomics of managed relocation. *Journal of the Association of Environmental and Resource Economists*, 3 (4):1023–1059, 2016.

L. E. Koehn, T. E. Essington, K. N. Marshall, W. J. Sydeman, A. I. Szoboszlai, J. A. Thayer, and E. Anderson. Trade-offs between forage fish fisheries and their predators in the California Current. *ICES Journal of Marine Science*, 74 (9):2448–2458, 2017. ISSN 1054-3139.

J. P. Kuriyama, Peter T.and Zwolinski, K. T. Hill, and P. R. Crone. Assessment of pacific sardine resource in 2020 for u.s. management in 2020 - 2021. Technical report, NOAA-Fisheries, 2020.

M. Maunder, P. Reilly, T. Tanaka, G. Schmidt, and K. Penttila. California Halibut Stock Assessment. Technical report, California Department of Fish and Wildlife, 2011.

R. D. Methot. Synthetic Estimates of Historical Abundance and Mortality for Northern Anchovy. *American Fisheries Society Symposium*, 6:66 – 82, 1989.

PFMC. Amendment 8 (to the northern anchovy fishery management plan) incorporating a name change to: The coastal pelagic species fishery management plan (appendix a). *Pacific Fishery Management Council*, 1998.

A. E. Punt, A. D. Maccall, T. E. Essington, T. B. Francis, F. Hurtado-Ferro, K. F. Johnson, I. C. Kaplan, L. E. Koehn, P. S. Levin, and W. J. Sydeman. Exploring the implications of the harvest control rule for Pacific sardine, accounting for predator dynamics: A MICE model. *Ecological Modelling*, 337(C):79–95, 2016. ISSN 0304-3800.

R. J. Reed and A. D. MacCall. Changing the size limit:how it could affect California halibut fisheries. *California Cooperative Oceanic Fisheries Investigations Reports*, 29:158 –166, 1988.

P. E. Rutquist and M. M. Edvall. Propt-matlab optimal control software. *Tomlab Optimization Inc*, 260(1):12, 2010.

J. N. Sanchirico and M. Springborn. How to get there from here: ecological and economic dynamics of ecosystem service provision. *Environmental and Resource Economics*, 48(2):243–267, 2011.
